# Age-Related Changes in Neuron–Microglia Interaction Mediated by Fractalkine Under Inflammatory Conditions

**DOI:** 10.3390/ijms262311378

**Published:** 2025-11-25

**Authors:** Rommy von Bernhardi, Franchesca Cortes, Claudia Narea, Claudia Metz, Gaston Godoy, Jaime Eugenin

**Affiliations:** 1 Faculty of Sciences, Universidad San Sebastian, Santiago 7510602, Chile; 2 Faculty of Chemistry and Biology, Universidad de Santiago de Chile, Santiago 9170022, Chile

**Keywords:** aging, CX3CL1, microglia, neurodegenerative disease, neuroinflammation, neuron-microglia interaction, TGFβ

## Abstract

Ageing results in an increased microglial activation and neuroinflammation. We are interested in assessing ageing-dependent changes in the amount and fractalkine (CX3CL1) proteoforms participating in neuron–microglia crosstalk that could be involved in microglia activation. We analysed age-dependent changes in CX3CL1, CX3CR1, and TGFβ mRNAs using RT-qPCR and CX3CL1 proteoforms using Western blot, in 3 to 20-month-old WT mice and an inflammatory mouse model (SRA^−/−^) treated with 0.5 mg/kg of intraperitoneal LPS, 2 ng of intrathecal TGFβ, or a vehicle. CX3CL1, CX3CR1, and TGFβ were affected by ageing. CX3CL1 mRNA was similar in young and adult mice but decreased by 52% in >20-month-old mice; adult mice showed a 3-fold increase in 70 kDa soluble CX3CL1. CX3CR1 showed a progressive increase, reaching a 2-fold increase in >20-month-old mice. TGFβ expression and cytokine reached their highest levels (3-fold increase) in adult mice and were reduced by 45% in >20-month-old mice. Inflammation, especially in SRA^−/−^ mice, produced an increase in CX3CL1 mRNA in adult mice and a maximal CX3CR1 mRNA level in old mice, which were nearly abolished by TGFβ. Our findings show age-related changes in CX3CL1 and TGFβ, with the highest levels observed in adult mice, an age at which the early mechanisms leading to neurodegenerative disease initiate.

## 1. Introduction

Ageing is a key risk factor for neurodegenerative diseases. Some common biological mechanisms participate in ageing and age-related diseases, with key roles played by inflammation [1]. Inflammation is associated with 10 of 14 of the best-described risk factors for dementia (i.e., smoking, hypertension, diabetes, hypercholesterolemia, obesity, excessive alcohol consumption, sedentarism, traumatic brain injury, depression, and pollution) [2,3].

### 1.1. Ageing-Related Changes in Neuroinflammation and Microglia Reactivity

The aged central nervous system (CNS) shows neuroinflammatory changes and an increased glial cell reactivity (reviewed in [4]). This chronic low-level neuroinflammation stimulates damage, increased oxidative stress, and lipid peroxidation, further increasing neuroinflammation [5,6]. Microglia activation affects neuron function [7,8] by increasing the production of inflammatory mediators, oxidative stress, and neurotoxicity, and inducing the release of soluble mediators by neurons and astrocytes [9,10] that affect synaptic function [11,12,13] and inter-neuronal connectivity, also impacting learning and memory.

Aged microglia show an increased inflammatory activation, characterised by the upregulation of MHC II, CD68, CD11b, CD14, and pattern recognition receptors [14,15,16]. This increased activation leads to the secretion of cytokines such as tumour necrosis factor α (TNFα), interferon γ (IFNγ), interleukin 6 (IL6), and interleukin 1β (IL1β) [4,17], chemokines like monocyte chemoattractant protein-1 (MCP1), reactive oxygen species (ROS), and nitric oxide (NO), all of which contribute to a neuroinflammatory environment. The level of transforming growth factor β1 (TGFβ1), a critical regulator of microglia activation, is also increased. However, the activation of downstream Smad3-TGFβ signalling, which has a vital role in the downregulation of inflammation and cytotoxicity, decreases, impairing its ability to downregulate microglial activation in the ageing brain [18].

### 1.2. Ageing-Related Changes in TGFβ Signalling

TGFβ1 is a key regulatory cytokine, attenuating inflammatory activation and oxidative stress and promoting neuroprotection [19,20]. TGFβ canonical signalling through the Smad pathway reduces the release of inflammatory cytokines, ROS, and NO [21,22]. Smad activation increases the transcription of MAP Kinase Fosfatase-1 (MKP-1), inactivating the phosphorylation of mitogen-activated protein kinases (MAPKs) and inhibiting inflammatory activation [21,23]. There is also activation of non-Smad-dependent TGFβ signalling pathways, MAPKs, and phosphatidylinositol 3-kinase (PI3K) [22,23,24].

The activation of Smad3 by inflammatory stimuli is reduced in ageing, affecting TGFβ functions [18]. The increased MAPK-mediated activation is potentiated by the reduced activation of the Smad pathway and further promotes inflammation, resulting in phenotypic and regulatory changes in glia, which shift from a homeostatic to a dysregulated activation state. The increased inflammatory activation in ageing can enhance cytotoxicity and further reduce Smad activation [23,24,25,26], promoting neurodegenerative changes. Increased TGFβ level appears also to promote the senescence of astrocytes and blood–brain barrier (BBB) dysfunction [27].

The TGFβ signalling-dependent microglial signature includes the downregulation of several genes in inflammatory microglia. Two genes that are especially relevant in the nervous system are the secreted protein acidic and rich in cysteine (*sparc*), involved in the regulation of microglia proliferation and microgliosis [28], and *cx3cr1*, which will be further discussed in Section 1.4.

### 1.3. Age-Related Changes in Neuron-Glia Interactions

Glia have a pivotal role in the preservation of functional neurons and participate in several brain diseases. Increased neuroinflammation promotes the dysregulation of microglia responses that have a critical role for the immune response [29] and the interaction with neurons mediated by the CX3CL1/CX3CR1 axis [30,31].

Understanding their interaction is a huge task given the complexity of the interaction of microglia with other cells in the brain. Although various microglia receptors and soluble mediators participate in microglia–neuron communication, we will concentrate on ageing-related changes in CX3CL1/CX3CR1 and TGFβ.

### 1.4. Regulation of Microglia by CX3CL1

CX3CL1 is a constitutively expressed neuronal transmembrane protein with four domains: the N-terminal chemokine domain (CKD), the mucin stalk domain (MS) that presents the CKD, the transmembrane domain (TM) involved in CX3CL1 aggregation to strengthen adhesion, and the cytosolic domain (CD) that anchors CX3CL1 to cytoskeletal proteins and increases cell adhesion [32,33]. TNFα and IFNγ can also induce CX3CL1 expression in astrocytes [33].

The 95 kDa transmembrane CX3CL1 (mCX3CL1) is cleaved into soluble CX3CL1 (sCX3CL1), containing CKD, by metalloproteases ADAM17/TACE [34] and ADAM10 [35] (65–70 kDa sCX3CL1) and cathepsin S [36] produced by microglia (40 kDa sCX3CL1) [37]. mCX3CL1 and sCX3CL1 bind to a single receptor coupled to G proteins (GPCRs), CX3CR1, expressed constitutively by microglia [38,39], serving a key role in microglia–neuron interaction [4,40,41].

Two signalling pathways mediate the activity of CX3CL1. One is an inflammatory pathway that activates NFκB, involving ERK, Jun N-terminal kinase (JNK), and p38 [42]. The second is an anti-inflammatory pathway mediated by the activation of AKT/ERK that induces the nuclear factor erythroid related factor 2 (Nrf2), increasing the transcription of antioxidant and cytoprotective genes, promoting the phagocytic and anti-inflammatory capacity of microglia [43,44,45].

The CX3CL1/CX3CR1 axis is proposed as an “off signal” that avoids the generation of a hostile environment for neurons [46,47]. The CX3CL1/CX3CR1 axis contributes to brain functions throughout life. In the adult brain, it regulates glutamatergic synaptic transmission and plasticity [48,49,50] and cognitive functions [51,52,53]. CX3CL1 appears to exert an anti-inflammatory function, reducing brain inflammation induced by the intracerebroventricular (ICV) injection of LPS [54] and inhibiting LPS-induced release of TNFα, IL6, and IL1β by microglia in culture [55,56]. In *cx3cr1*^−/−^ mice, microglial IL1β expression, neurotoxicity, and mortality induced by intraperitoneal (i.p.) LPS injections are higher than those observed in *cx3cr1^+/−^* mice [57].

### 1.5. Regulation of Microglia by CX3CL1 in Ageing

It has been reported that CX3CL1 and CX3CR1 are reduced in aged mice, being associated with an increased microglial cell activation [58]. Mice 18 to 22 months old have reduced CX3CL1 in the cortex and hippocampus. In addition, CX3CL1 remains unchanged 24 h after i.p. LPS injection, compared with the effect reported in 3–6-month-old mice, and CX3CR1 mRNA diminishes 4 h after i.p. LPS in aged and adult mouse microglia. In aged mice, LPS induced a persistent (24 h) downregulation of CX3CR1, with microglial activation and a reduced TGFβ expression [59]. Those changes could lead to an enhanced inflammation, deficits in synaptic remodelling, and eventually to cognitive impairment. CX3CL1 treatment reduces age-related microglial cell activation [58].

Diverse functions, often contradictory, have been proposed for mCX3CL1 and sCX3CL1 [60]. APP/PS1 mice deficient in CX3CL1 show less Aβ deposition, but increased Tau phosphorylation in neurones [30]. Recombinant sCX3CL1 did not affect Aβ deposition and Tau phosphorylation, indicating that mCX3CL1 participates in both processes [60]. CX3CL1 appears to be also involved in the attenuation of an inflammatory phenotype in a Tauopathy mouse model [61]. However, a specific cleavage variant of sCX3CL1 is needed to reduce Tau pathology in AD [62], indicating that either the various forms of CX3CL1 have different pharmacodynamic properties, or other signalling pathways are also involved in the protective effect.

CX3CL1 also has diverse effects on neuroinflammation and neurodegeneration in α-synuclein models of PD [63]. It can be neuroprotective, inhibiting dopaminergic cell death in the substantia nigra (SN) and striatum and motor impairment in rats treated with the recombinant adeno-associated virus (rAAV) coding for α-synuclein [64]. Microgliosis, neuroinflammation, and dopaminergic neuronal death in the SN induced by human α-synuclein overexpression are similar in *cx3cr1*^+/+^ and *cx3cr1*^−/−^ mice. However, human α-synuclein A53T overexpression induces an exacerbated neurodegeneration compared with the human α-synuclein overexpression and enhanced neurodegeneration in *cx3cr1*^−/−^ mice [65].

In the 1-methyl-4-phenyl-1,2,3,6-tetrahydropyridine (MPTP) Parkinson’s disease (PD) mouse model, the deletion of *cx3cl1* or *cx3cr1* aggravates the dopaminergic neuronal loss and increased microglial activation [57], but the SN injection of recombinant sCX3CL1 in CX3CL1-null mice ameliorated MPTP-induced microglial activation and inflammation, reducing dopaminergic neuronal death and motor impairment; however, treatment with a mutant mCX3CL1 resistant to cleavage has no effect on the MTPT-induced PD phenotype [66]. CX3CR1 deficiency results in microglia activation and increased neurodegeneration after LPS injections in PD and amyotrophic lateral sclerosis (ALS) mouse models [43,67] and worsens Alzheimer’s disease (AD)-related neuronal deficits associated with microglial activation and elevated chemokines [43]. However, CX3CR1 deficiency results in an increased Aβ clearance and prevents neuron loss in other AD mouse models [68].

Thus, for the multiple functions involving the CX3CL1/CX3CR1 axis, contradictory results have been often obtained regarding its effect and regulation. Here, we study how ageing and inflammatory conditions influence the mRNA and protein levels for CX3CL1 (40- and 70 KDa), CX3CR1, and TGFβ using young (3–7-month-old), adult (12–15-month-old), and old (older than 20 months) mice. Acute inflammation was generated by the administration of LPS for 1 h, whereas chronic inflammatory conditions involved the use of scavenger receptor A knockout (SRA^−/−^) mice. The use of an acute treatment with LPS allowed us to evaluate the direct effect of the original inflammatory stimuli and not the cascade of endogenous activation and regulatory responses elicited by a prolonged stimulation. We found that ageing induces changes in the levels of mRNA and protein of the CX3CL1/CX3CR1 axis, and conditions the way neurons and glia respond to acute and chronic inflammatory stimuli.

## 2. Results

### 2.1. Age-Related Changes for CX3CL1, CX3CR1, and TGFβ

**Figure 1:**
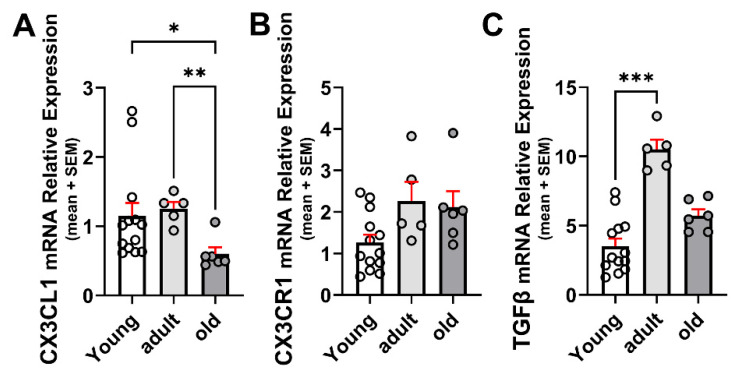
Age-related changes in mRNA for CX3CL1, CX3CR1, and TGFβ in the brain. (**A**) CX3CL1 mRNA relative expression to β-Actin in young (3–7-month-old), adult (12–15-month-old), and old (older than 20 months) WT mice under control (unstimulated) condition (n = 13, 6, and 6, respectively). CX3CL1 mRNA was similar in young and adult WT mice, decreasing by 52% in mice older than 20 months. (**B**) CX3CR1 mRNA relative expression to β-Actin at different ages; n = 13, 6, and 6, respectively. CX3CR1 mRNA increased not significantly in adult WT mice and persisted on a similar level in old mice. (**C**) TGFβ mRNA relative expression to β-Actin. TGFβ increased by 3-fold in adult mice but decreased in mice older than 20 months of age. N = 13, 6, and 6 for the different age groups, respectively: bars correspond to means and dispersion of data is expressed as standard error of the mean (SEM). Statistical analysis was performed by a Kruskal–Wallis followed by Dunn’s post hoc test. (**A**) *p* = 0.0055; (**B**) *p* = 0.0627; (**C**) *p* = 0.0007. *: *p* < 0.05; **: *p* < 0.01; ***: *p* < 0.001.

CX3CL1 mRNA levels were similar in the brains of young and adult wild-type (WT) mice, decreasing by 52% in mice older than 20 months (Figure 1A). Although the change did not reach statistical significance, the CX3CR1 mRNA level was on average about 78% greater in adult than in young mice and persisted at similar levels in old mice (*p* = 0.06; Figure 1B).

TGFβ increased significantly by 3-fold in the brains of 12–15-month-old mice compared with 3–5-month-old mice and decreased by 46% in mice older than 20 months, although remaining 60% higher than that observed in young mice (Figure 1C). The increased TGFβ mRNA is consistent with previous reports on the concentration of TGFβ in adult mice brains [18].

**Figure 2:**
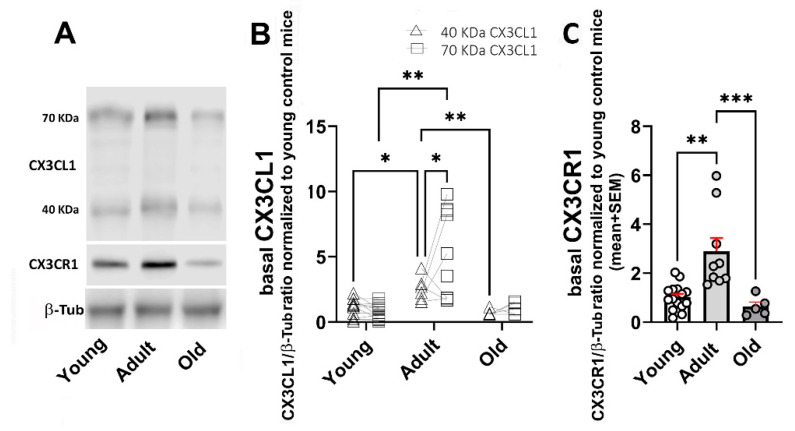
Age-related changes in protein levels for 40 and 70 KDa sCX3CL1 and CX3CR1 in the brain. (**A**) Western blot of brain lysates obtained from young (3–7-month-old); adult (12–15-month-old), and old (older than 20 months) WT mice under control (unstimulated) condition. Immunodetection of sCX3CL1 (40- and 70 kDa proteoforms) and CX3CR1 are shown. Beta-tubulin (β-Tub; 55 kDa) was used as loading control. Adult WT mice showed an increased amount of 40 and 70 kDa sCX3CL1, with predominance of the 70 kDa proteoform. (**B**) CX3CL1/β-Tub ratio normalized to young untreated mice, for 40 kDa (n = 12, 6, and 5) and 70 kDa (n = 14, 9, and 6, for young, adult, and old mice, respectively), is compared for each age group, respectively. (**C**) CX3CR1/β-Tub ratio normalized to young untreated mice; bars, means; red vertical lines, standard error of the mean (SEM); n = 15, 9, and 5, respectively. CX3CR1 also exhibited an increased level in adult mice. Young mice were 3–7 months old; adult mice were 12–15 months old; and old mice were older than 20 months. Statistical analysis in (**B**) was performed using a Two-way mixed ANOVA followed by Tukey’s post hoc test. Two-way mixed ANOVA revealed a significant effect of both ageing and type of proteoform of sCX3CL1 upon the basal levels of 40 and 70 kDa sCX3CL1 with [F (1.048, 24.10) = 21.78; *p* < 0.0001] and [F (1, 46) = 4.558; *p* = 0.0381], respectively. Statistical analysis of CX3CR1 data in C was performed using a Kruskal–Wallis (*p* = 0.0003) followed by Dunn’s post hoc test. Asterisks in (**B**,**C**): *: *p* < 0.05; **: *p* < 0.01; ***: *p* < 0.001.

Adult WT mice showed an increased amount of 40 and 70 kDa sCX3CL1 compared with young animals (a 2.3- and 7.1-fold increase, respectively). In contrast to what was observed in young mice, the 70 kDa proteoform was predominant in adult mice. In mice older than 20 months, the levels of 40 and 70 kDa sCX3CL1 decreased to levels like those observed in young mice (Figure 2A,B). CX3CR1 also showed an increased concentration in adult mice, with a 2.8-fold increase compared with young mice, which was reduced by 78% in mice older than 20 months, lower by 37% than in young mice (Figure 2A,C).

### 2.2. Age-Dependent Changes on the Regulation of mRNAs for CX3CL1, CX3CR1, and TGFβ by Inflammatory Stimulation

**Figure 3:**
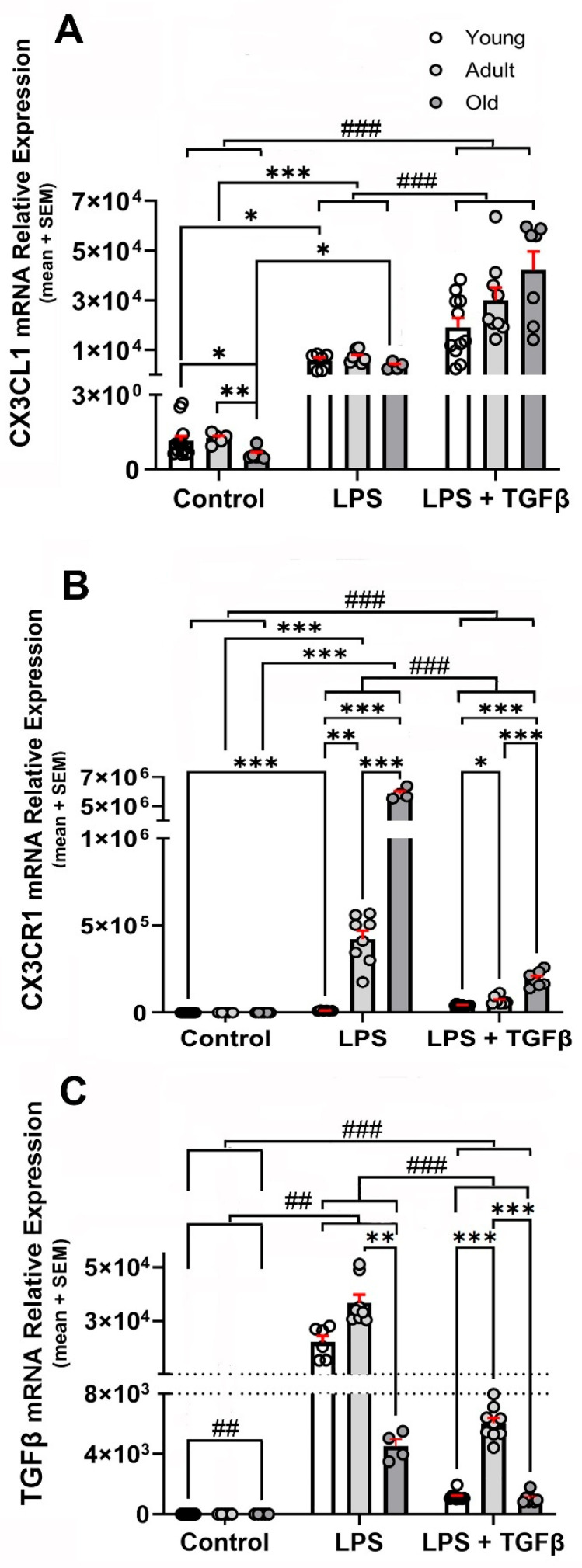
LPS- and LPS + TGFβ-dependent changes in the expression of CX3CL1, CX3CR1, and TGFβ mRNA in the brain. CX3CL1, CX3CR1, and TGF mRNA relative expressions to β-Actin (loading control) were analysed in young, adult, and old WT mice under control (unstimulated) condition (n = 13, 6, and 6, respectively) and after stimulation with i.p. LPS (0.5 mg/kg) injection (n = 6, 8, and 5, respectively) and i.p. LPS + i.t. TGFβ (2 ng) injections (n = 11, 9, and 7, respectively); data were expressed as mean (bars) + SEM (vertical red lines); (**A**) inflammatory stimulation increased manyfold CX3CL1 mRNA expression, with the highest expression observed in adult mice. TGFβ potentiated the inflammation-induced expression of CX3CL1. Young mice were 3–7 months old; adult mice were 12–15 months old; and old mice were older than 20 months. Statistical analysis was performed by a Two-way mixed ANOVA followed by Tukey’s post hoc test. Fixed effects (type III) did not revealed significant effect for age variable [*p* < 0.0800; F (1.972, 59, 17) = 2.646], but it did for treatment with LPS and TGFβ [*p* < 0.0001; F (2, 60) = 61.88] and age x treatment with LPS and TGFβ [*p* = 0.0088; F (4, 60) = 3.740]. (**B**) CX3CR1 mRNA relative expression to β-Actin, was analysed in young, adult, and old WT mice under control (unstimulated) condition (n = 13, 6, and 6, respectively) and after stimulation by i.p. LPS (0.5 mg/kg) injection (n = 6, 8, and 5, respectively) and i.p. LPS + i.t TGFβ (2 ng) injections (n = 11, 9, and 7, respectively); inflammatory stimulation induced an increase in CX3CR1 mRNA. Co-treatment with TGFβ (2 ng) reduced the LPS-induced increase. Data were expressed as mean (bars) + SEM (vertical red lines); young mice were 3–7 months old; adult mice were 12–15 months old; and old mice were older than 20 months. Two-way mixed ANOVA revealed a significant effect of both age [*p* < 0.0001; F (1.390, 21, 54) = 2691] and treatment with LPS and TGFβ [*p* < 0.0001; F (2, 29) = 2205]. Multiple comparisons were performed with Tukey’s post hoc test; (**C**) TGFβ mRNA relative expression to β-Actin was analysed in young, adult, and old WT mice under control (unstimulated) condition (n = 13, 6, and 6, respectively) and after stimulation by i.p. LPS (0.5 mg/kg) injection (n = 6, 8, and 5, respectively) and i.p. LPS + i.t 2 ng TGFβ injections (n = 11, 9, and 7, respectively); Data were expressed as mean (bars) + SEM (vertical red lines). TGFβ levels were the highest in adult WT mice. Inflammatory activation resulted in a robust increase in TGFβ expression that was also maximal in adult mice. Young mice were 3–7 months old; adult mice were 12–15 months old; and old mice were older than 20 months. Two-way mixed ANOVA revealed significant effect of age [*p* < 0.0001; F (1.116, 32.93) = 60.94] and treatment with LPS and TGFβ [*p* < 0.0001; F (2, 59) = 205.4]. Multiple comparisons were performed with Tukey’s post hoc test. *: *p* < 0.05; **: *p*< 0.01; ***: *p*< 0.001. Significant differences between inflammatory conditions observed for all age groups are represented by “#”: ##: *p* < 0.01; ###: *p* < 0.001.

Inflammatory stimulation with i.p. LPS injection in WT mice resulted in a ≈5500-fold increase in CX3CL1 mRNA expression compared with the basal expression for each age group (Figure 3A).

TGFβ potentiated the inflammation-induced expression of CX3CL1 by 3.4-fold in young, 4.2-fold in adult, and 11.6-fold in old mice, respectively, although the amount of CX3CL1 mRNA observed in response to the LPS + TGFβ stimulation did not show statistical significant differences among the three age groups (Figure 3A).

Inflammatory stimulation with i.p. LPS injection in WT mice induced an increase in CX3CR1 mRNA expression that showed an ageing-dependent potentiation (Figure 3B). An 886-fold increase was observed in young mice, but the expression was increased by 1866-fold in adult and by 2591-fold in old mice. A co-treatment with TGFβ resulted in a reduction of more than 95% of the LPS-induced increase, although the level of expression remained significantly higher (*p* < 0.001) than that observed in unstimulated mice of the same age group, with an increase of 41.7-fold in young, 30.5-fold in adult, and 91.2-fold in old WT mice (Figure 3B). As will be discussed in the Section 3, the increased expression of CX3CR1 mRNA only resulted in a discrete increase in the receptor.

TGFβ mRNA levels were highest in adult WT mice, increasing 3-fold in adults and 1.6-fold in old mice compared with young WT mice, respectively (Figure 1C). Inflammatory activation induced by i.p. LPS injection resulted in a robust increase in TGFβ expression levels, with TGFβ mRNA showing its maximal expression in adult mice and decreasing by 88% in old mice. A co-treatment with an i.t. TGFβ injection resulted in a conspicuous reduction in the TGFβl mRNA induced by LPS. The reduction induced by TGFβ was 95% for young, 84% for adult, and 77% for old mice, respectively (Figure 3C).

### 2.3. Age-Dependent Changes in sCX3CL1 and CX3CR1 in Response to Inflammation and TGFβ

**Figure 4:**
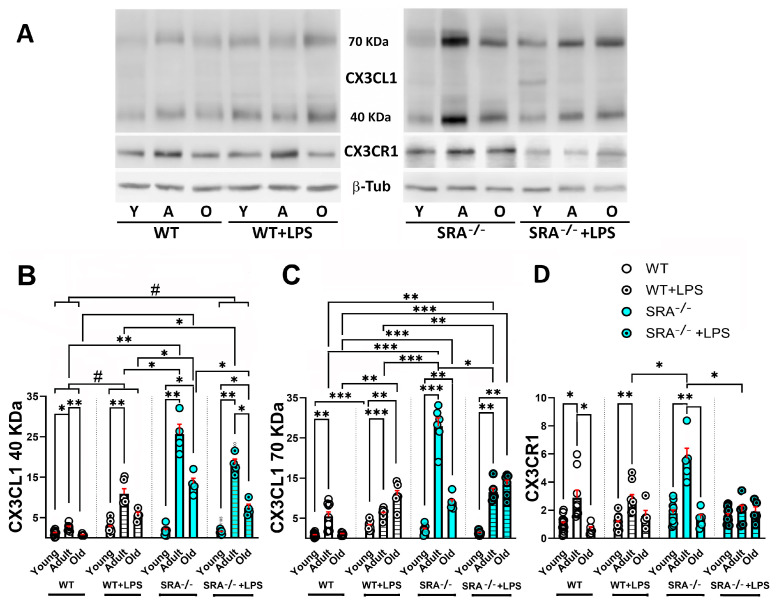
Age-dependent changes in sCX3CL1 and CX3CR1 protein levels induced by LPS in the brains of WT and SRA^−/−^ mice. (**A**) Western blot of brain lysates obtained from young (3–7-month-old); adult (12–15-month-old), and old (older than 20 months) WT and SRA^−/−^ mice under control (unstimulated) condition or stimulated with i.p. LPS (0.5 mg/kg) injection (WT + LPS and SRA^−/−^ + LPS). Immunodetection of sCX3CL1 (40- and 70 kDa proteoforms) and CX3CR1 are shown; β-Tub was used as loading control. (**B**,**C**) sCX3CL1 β-Tub ratio normalized to young untreated mice, for 40 and 70 kDa, respectively, is compared for the different stimulation conditions for each age group; data were expressed as mean (bars) + SEM (vertical red lines); n = 6 to 14 mice per condition. Inflammation resulted in an increased amount of soluble proteoforms of CX3CL1. The increment of 40 kDa sCX3CKL1 was maximal in adult mice, whereas a decrease in sCX3CL1 was observed in old mice. (**D**) CX3CR1/β-Tub ratio normalized to young untreated mice, expressed as mean (bars) + SEM (vertical red lines); n = 6 to 15 mice per condition. SRA^−/−^ mice, which present a basal inflammatory activation, showed the most robust increase in CX3CL1 and CX3CR1, whereas the stimulation of SRA^−/−^ mice with LPS resulted in a decreased induction of sCX3CL1 and CX3CR1. Young mice were 3–7 months old; adult mice were 12–15 months old; and old mice were older than 20 months. Statistical analysis was performed using a Two-way mixed ANOVA followed by Tukey’s post hoc test. For the 40 kDa sCX3CL1, there was a significant effect of age [*p* < 0.0001; F (1.515, 47, 73) = 292.5] and inflammatory condition (LPS or SRA^−/−^) [*p* < 0.0001; F (3, 63) = 126.5]. Similarly, for the 70 kDa sCX3CL1, Two-way mixed ANOVA also revealed significant effects of age [*p* < 0.0001; F (1.578, 31, 57) = 47.65] and inflammatory condition [*p* < 0.0001; F (3, 46) = 9.086]. Multiple comparisons were performed with Tukey’s post hoc test; *: *p* < 0.05; **: *p* < 0.01; ***: *p* < 0.001. Significant differences between inflammatory conditions observed for all age groups are represented by “#”: #: *p* < 0.05.

Inflammation after treatment with i.p. LPS injection or in unstimulated SRA^−/−^ mice resulted in an increased amount of soluble proteoforms of CX3CL1 compared with control WT mice (Figure 4A,B). The increment of 40 kDa sCX3CKL1 was maximal in adult mice, with a 4.3-fold increase after LPS stimulation and 10-fold increase in SRA^−/−^ mice (Figure 4B).

For the 70 kDa sCX3CL1 proteoform, the maximal increase was observed in SRA^−/−^ mice, with a 5.1-fold increase. However, in WT mice and SRA^−/−^ mice exposed to LPS, the maximal induction of 70 kDa sCX3CL1 was observed in old mice. Furthermore, the treatment of SRA^−/−^ mice with LPS resulted in a reduction of 70 KDa sCX3CL1, especially conspicuous in adult mice (Figure 4C). A decrease in sCX3CL1 was observed in old mice, although the level was higher than that observed in unstimulated young mice (Figure 4B,C). For CX3CR1, there is no increase after LPS stimulation for any of the age groups, although a 2-fold increase was observed in adult SRA^−/−^ mice. SRA^−/−^ + LPS showed CX3CR1 levels that were lower than those observed in SRA^−/−^ mice, like the unstimulated group (Figure 4D). Thus, SRA^−/−^ mice, which present a basal chronic inflammatory activation, showed the most robust increase in CX3CL1 and CX3CR1, resulting in increased amounts of CX3CR1 compared with WT mice stimulated with i.p. LPS (*p* < 0.001). The stimulation of SRA^−/−^ mice with LPS resulted in a decreased response of sCX3CL1 and CX3CR1 compared with the amount of sCX3CL1 and CX3CR1 observed in unstimulated SRA^−/−^ mice, in agreement with the previous results showing a decreased inflammatory activation by LPS in glia from SRA^−/−^ mice [15,69,70].

**Figure 5:**
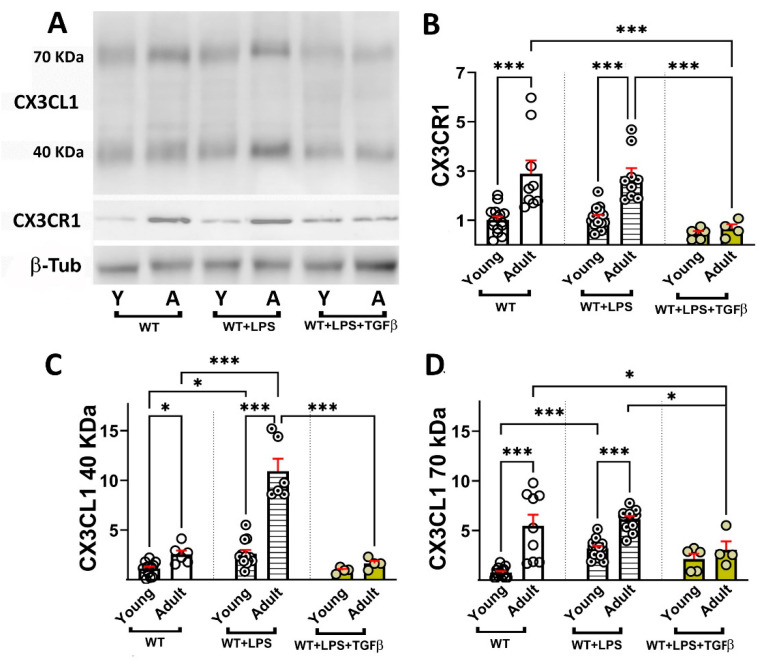
Effect of TGFβ on age-dependent changes in sCX3CL1 and CX3CR1 protein levels induced by LPS. (**A**) Western blot of brain lysates obtained from young (3–7-month-old) and adult (12–15-month-old) WT mice under control conditions (unstimulated), or stimulated with i.p. LPS (0.5 mg/kg) injection (WT + LPS) or i.p. LPS and i.t. TGFβ (2 ng) injections (WT + LPS + TGFβ). Immunodetection of sCX3CL1 (40- and 70 kDa proteoforms) and CX3CR1 are shown; β-Tub was used as loading control. (**B**) CX3CR1/β-Tub ratio normalized to young untreated mice, expressed as mean (bars) + SEM (vertical red lines); n = 5 to 15 mice per condition. TGFβ i.t. injection resulted in a significant decrease in CX3CR1 in young and adult mice. (**C**,**D**) sCX3CL1/β-Tub ratio normalized to young untreated mice, for 40 and 70 kDa, respectively, is compared after different stimulation conditions for each age group, expressed as mean (bars) + SEM (vertical red lines); n = 5 to 14 mice per condition. TGFβ abolished the age-related and LPS-induced increase in both sCX3CL1 proteoforms. Young mice were 3–7 months old and adult mice were 12–15 months old. Statistical analysis was performed using a Two-way mixed ANOVA followed by Tukey’s post hoc test. For the 40 kDa sCX3CL1, significant effects were obtained for age [*p* < 0.0001; F (1, 38) = 54.58] and for inflammatory condition [*p* < 0.0001; F (2, 38) = 63.41]. For the 70 kDa sCX3CL1, significant effects of age [*p* < 0.0001; F (1, 51) = 35.50] and inflammatory condition [*p* = 0.0010; F (2, 51) = 7.917] were observed upon the levels of 40 and 70 kDA sCX3CL1. Statistical analysis also demonstrated significant effects of age [*p* < 0.0001; F (1, 51) = 23.98] and inflammatory condition [*p* = 0.0003; F (2, 51) = 9.797] on CX3CR1 levels in brain homogenates. Multiple comparisons were performed with Tukey’s post hoc test. *: *p* < 0.05; ***: *p* < 0.001.

Adult mice showed a similar age-dependent increase in CX3CR1 in unstimulated and LPS-stimulated mice of 2.8- and 2.5-fold, respectively (Figure 5A,B), indicating that LPS treatment did not induce CX3CR1 in young and adult mice. However, i.t. TGFβ injection resulted in a decrease in CX3CR1 by 58% in young and by 76% in adult mice (Figure 5B).

An age-dependent increase in sCX3CL1 was observed, which was 2.3-fold for the 40 and 7.1-fold for the 70 kDa proteoforms (Figure 5C,D). LPS increased sCX3CL1 at both ages, although the induction of 40 kDa sCX3CL1 was more robust for adult (4.2-fold) than for young mice (2.3-fold). In contrast, LPS induced a robust increase (4.2-fold) in 70 kDa sCX3CL1 in young mice and lacked induction with respect to untreated adult mice (1.1-fold). TGFβ abolished the age-related and LPS-induced increase in both sCX3CL1 proteoforms, which exhibited similar levels to those observed in unstimulated young mice (Figure 5C,D).

It is interesting that, although TGFβ resulted in a robust potentiation of CX3CL1 mRNA, as shown in Figure 3C, the effect at the protein level, as observed in Figure 5C,D, was to abolish the production of both 40 kDa and 70 kDa sCX3CL1, an effect that is coherent with the regulatory effect of TGFβ on inflammation [21,22,23,71,72,73] given that the activation of both metalloproteases 10 and 17 and Cathepsin S are induced under inflammatory conditions, promoting further neuroinflammation [40,74,75,76,77]. On the contrary, for CX3CR1, both at the expression level, as shown in Figure 3B, and the protein level, as shown in Figure 5, TGFβ reduced the induction of CX3CR1 observed under inflammatory stimulation by LPS [78] in contrast with previous reports [79].

## 3. Discussion

**Figure 6:**
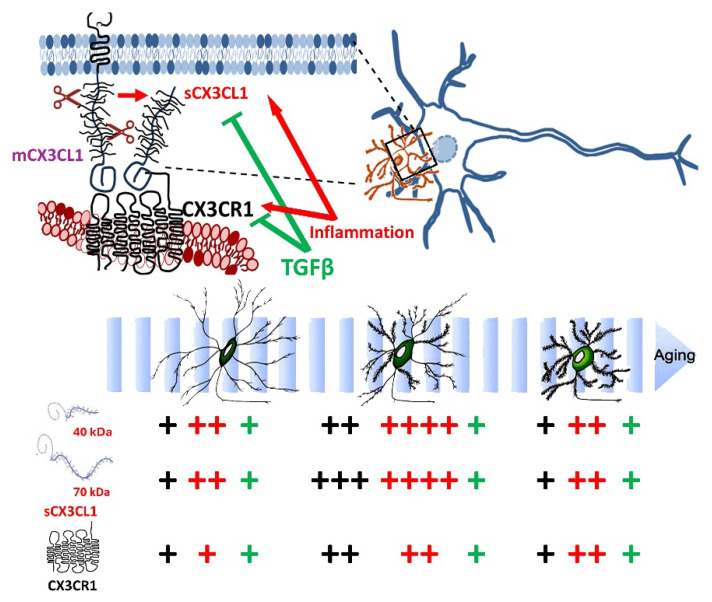
Working model for the ageing-related changes in CX3CL1/CX3CR1 regulation. The CX3CL1 pathway is critical for neuron–microglia crosstalk and the regulation of microglial activation. The number of “+” shows the abundance of CX3CL1 and CX3CR1 at the different age groups; in black, the control unstimulated condition, in red, the effect of inflammatory conditions (LPS treatment and SRA^−/−^ mice); in green, the regulation by TGFβ on the effect inflammation.

Age-related changes in CX3CL1 mRNA showed the highest levels in adult mice, followed by a CX3CL1 mRNA reduction in old mice (older than 20 months). The changes in CX3CL1 mRNA showed a similar profile to the levels reached by sCX3CL1 proteins. By contrast, despite the CX3CR1 protein exhibiting a similar increase in adults followed by a decrease in old mice, such changes in the protein were not accompanied by a significant variation in CX3CR1 mRNA. Interestingly, the increase in adult sCX3CL1 favoured the expression of the 70 kDa proteoform (Figure 6). We focused on 12–15-month-old mice [18], equivalent to 50-year-old humans, because, as we have previously discussed [78], neurodegenerative diseases such as AD develop over two to three decades in humans. Thus, the causal pathophysiological changes leading to neurodegenerative disease in older persons start when the person is 50–55 years old.

The age-related changes could depend on age-dependent changes in CX3CL1 processing related to changes in the amount, activity, or enzymatic efficiency of proteases involved in the cleavage of mCX3CL1 [80,81]. ADAM-10, ADAM-17, and Cathepsin S participate in the native 95 kDa mCX3CL1 cleavage from the neuronal membrane [77,82]. ADAM10 and ADAM17 are well studied metalloproteases, involved in the processing of numerous membrane-associated proteins, including CX3CL1 [83,84,85]. ADAM17/TACE [34] and ADAM10 [35] cleave mCX3CL1, generating a 65–70 kDa soluble CX3CL1 (sCX3CL1) that contains the N-terminal CKD. Aged rats show a reduced ADAM 17 activity, which could contribute to the reduction in 70 KDa proteoforms observed in older mice. The age-dependent decrease in activity is associated with an impaired response in aversive memory assays [86].

Cathepsin S is a cysteine lysosomal protease released in response to neurotrophic and inflammatory mediators. The amount of Cathepsin S shows age-dependent changes. An age-dependent upregulation of immature and mature forms of Cathepsin S is observed in 6-month-old mice [82]. DNA microarrays show that Cathepsin S gene expression in 25-month-old mice is increased compared to 5-month-old mice [87]. Although we expected to observe increased amounts of 40 kDa sCX3CL1 with ageing, there was a mild increase of 40 kDa in adult mice, but the most conspicuous age-related increase in sCX3CL1 was the 70 kDa proteoform. Both proteoforms diminished in old mice, in the age range when Cathepsin S is upregulated.

There are also reports that the effect of CX3CL1 on microglia isolated from 15-month-old rats differs from the response of microglia obtained from young animals. Although microglia obtained from mice at various ages respond to inflammatory conditions, like LPS, in a similar fashion, their response after treatment with CX3CL1 shifts towards an increased inflammatory activation, with a bi-phasic, U-shaped concentration response curve for both CX3CL1 peptides, showing a decreased anti-inflammatory effect at low concentrations of sCX3CL1 [88]. Picomolar CX3CL1 is anti-inflammatory, but CX3CL1 at nanomolar concentrations becomes proinflammatory. The anti-inflammatory response of low-concentration CX3CL1 is significantly reduced in aged cells, responding with increased TNFα release compared to microglia from young rats [88]. The binding of full sCX3CL1 and CKD sCX3CL1 exhibited a similar affinity for CX3CR1. These observations suggest that the amount, and not necessarily the CX3CL1 proteoform, can be a determinant of the functional effect of activating the CX3CL1/CX3CR1 axis, and further analysis should be undertaken with a quantitative approach.

In this study, we observed the highest levels of CX3CL1 mRNA and sCX3CL1 proteoforms in adult 12–15-month-old mice. We also evaluated the effect of a short acute LPS exposure to assess the direct impact of inflammation, avoiding the development of the complex response of mice to prolonged inflammatory stimuli. Short-lived inflammation induced a conspicuous increase in CX3CL1 and CX3CR1 mRNA that only induces a discrete increase in CX3CL1 and CX3CR1 proteins, with the most conspicuous response in adult mice. TGFβ resulted in a potentiation of the induction of CX3CL1; by contrast, the induction by LPS of CX3CR1 and TGFβ was reduced by TGFβ treatment. At the protein level, TGFβ reduced both 40 kDa and 70 kDa sCX3CL1 and CX3CR1, suggesting that the regulation of inflammation decreases the activation of the CX3CR1/CX3CR1 axes (Figure 6). Our experiments with SRA^−/−^ mice, which present an inflammatory phenotype under basal conditions [15,69,70], resulted in an induction of sCX3CL1 and CX3CR1 of greater magnitude, indicating that a sustained inflammation involving an endogenous neuroinflammatory response may participate in the regulation of the CX3CL1 axis (Figure 6). Sustained microglial cell activation and neuroinflammation are relevant in neurodegenerative conditions. At the initial stages of AD, there are increased numbers of microglia and astrocytes due to the sustained cellular proliferation in response to disturbances, loss of homeostasis, or proteostasis in the affected brain territories [89,90]. Increases in dysregulated microglia, with beading and fragmented processes, are reported in several neurodegenerative diseases [91], indicating the change in microglia towards a senescent or disease-associated type [89,92,93], which contributes to cytotoxic changes in the brain [92,94,95].

The differences in the inflammatory regulation exerted by mCX3CL1 and sCX3CL1 are still an open question. It is proposed that the binding of mCX3CL1 to CX3CR1 acts as a cell–cell adhesion molecule with an anti-inflammatory effect, maintaining microglia in a homeostatic state [30,31,39,96]. By contrast, sCX3CL1 could promote the inflammatory activation of microglia, resulting in chemoattraction and the increased reactivity of microglia [37]. The apparent dual pro- and anti-inflammatory effects participate in neuronal migration, synaptic pruning, and synaptic maturation during development, maintaining homeostasis and regulating the balance between pro- and anti-inflammatory cytokines in response to the microenvironment in the adult brain. However, this clear-cut binary response has been elusive to find. A key question regarding the role of CX3CL1/CX3CR1 signalling for microglia is whether the binding to CX3CR1 by mCX3CL1 compared to one of the sCX3CL1 proteoforms impacts their regulation differently. Differential functions for mCX3CL1 and sCX3CL1 are reported also in the APP/PS1 mouse model, with cx3cl1^−/−^ APP/PS1 mice exhibiting a reduced Aβ deposition but an enhanced Tau phosphorylation in neurones. Surprisingly, the effects on Aβ and Tau were unmodified by the transgenic expression of sCX3CL1, suggesting that mCX3CL1 engaged in both processes [30].

The conflicting results regarding the anti- vs. proinflammatory properties of different CX3CL1 proteoforms can be associated with the existence of other conditions that may modify the regulatory effect of CX3CL1. Evidence showing the divergent effects of CX3CL1 on activated microglia depending on its concentration and the age of the individual appears to be especially appealing for our results. The anti-inflammatory effect of CX3CL1 is observed at thousand-fold smaller concentrations than the concentration of proinflammatory CX3CL1; the anti-inflammatory response of low-concentration CX3CL1 is significantly reduced in aged cells, responding with an increased TNFα release compared to microglia from young rats [88]. The data suggest that aged microglia are less sensitive to the homeostatic signalling of low concentrations of CX3CL1, while simultaneously more sensitive to the proinflammatory signalling of high concentrations of CX3CL1 in rat and mouse models [88]. We found thousand-fold and higher increases in adult mice exposed to inflammatory conditions compared with young mice. There is also a significant difference depending on the inflammation mechanism, suggesting that the context in which microglia are exposed to the various stimuli that they encounter in their environment is significant. Ageing induces changes in the CX3CL1/CX3CR1 axis at the mRNA and protein level, and conditions the way it responds to acute and chronic inflammatory stimuli.

## 4. Materials and Methods

### 4.1. Animals and Animal Protocols

Founders for the WT (C57B6) and SRA^−/−^ (B6.Cg-Msr1tm1Csk/J) mice (Table 1) were obtained from Jax Mice (Jackson Laboratory, Bar Harbor, ME, USA) and their developer Dr. Tatsuhiko Kodama, who originally developed SRA^−/−^ in a 129/ICR background (Research Center for Advanced Science and Technology, University of Tokyo, Tokyo, Japan), and were kept at the institutional animal facility of the Medical Research Center, Pontificia Universidad Católica de Chile School of Medicine. Mice were housed at a maximum density of five adult mice per cage, in a temperature-regulated room with a 12 h light/dark cycle and with free access to food and water. All procedures were performed following the animal handling and bioethical requirements defined by the Pontificia Universidad Católica de Chile School of Medicine Ethics Committee, where the mouse colony was housed, The Ethics Committee on the Care and Use of Animals in Research at Universidad San Sebastián, Chile, and the National Institutes of Health guide for the care and use of Laboratory animals (NIH Publications No. 8023, revised 1978).

**Figure 7:**
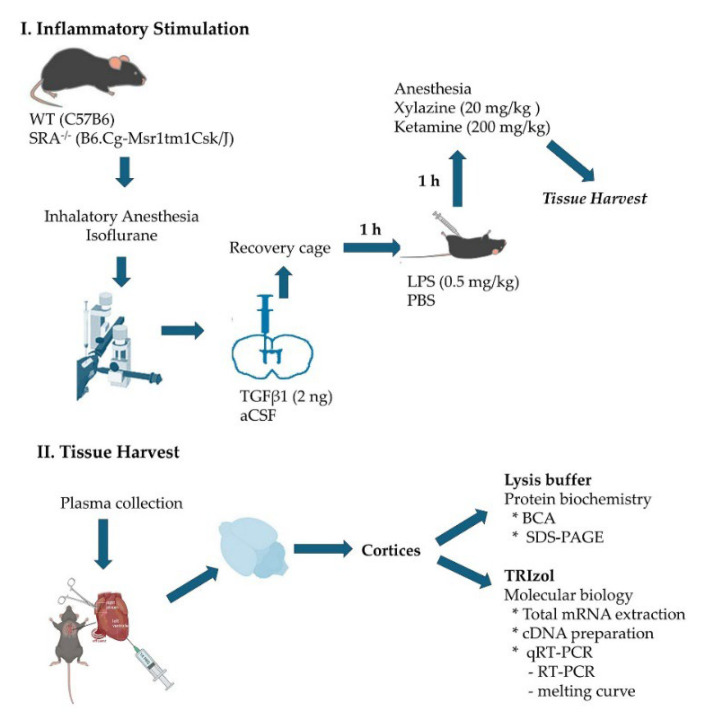
Experimental procedures and tissue harvest protocol.

Figure 7 provides a schematic description of the experimental procedures and tissue harvest protocol. All mice followed the same protocol, receiving an injection of the inflammatory mediator or vehicle, depending on the experimental condition.

- Stereotaxic intracerebroventricular TGFβ injection

Mice were anesthetized in a chamber with a 3 L/min flow of O_2_ containing 4% isoflurane. Once the state of unconsciousness was verified by interdigital stimulation, mice were positioned in the stereotaxic apparatus, maintaining anesthesia through a mouse mask. The skull was secured to the stereotaxic apparatus through the ear using the graduated bars. A 1.5 cm anteroposterior sagittal incision was made. With the Hamilton syringe positioned exactly at the Bregma, the anteroposterior and lateral coordinates were recorded. For ICV injections, the coordinates used were 1 mm medial–lateral, 0.3 mm anteroposterior, and 2.5 mm dorsoventral. The Hamilton syringe was loaded with 2 μL of 2 ng TGFβ1 that was injected at a rate of 1 μL/min.; the same volume was used for the vehicle control, artificial cerebrospinal fluid (aCSF). After the injection and a 2 min wait, the syringe was removed. The mouse was taken from the stereotaxic apparatus, the wound was closed, and the animal was placed in a warm recovery cage under observation, according to animal welfare guidelines.

- Generation of acute systemic inflammation

One hour after intrathecal drug injections, mice received an i.p. injection of 0.5 mg/kg of LPS from *Escherichia coli* O55:B5 (437627, Merck, Rahway, NJ, USA) or PBS as a vehicle. Mice were maintained for 1 h with water and food ad libitum before perfusion.

- Obtention of the tissue

One hour after i.p. LPS or vehicle injection, mice were deeply anesthetized with a mixture of 20 mg/kg of Xylazine and 200 mg/kg of Ketamine. A blood sample was drawn into heparin-lined tubes for plasma collection, and mice were transcardially perfused with 40 mL of ice-cold physiological solution (0.9% NaCl and 0.5% NaNO_2_ in dH_2_O) to proceed in obtaining the brain, removing meninges, cerebellum, and brain stem, and harvesting the cortices, generating homogenate samples for protein biochemistry and molecular biology analysis. Brain homogenates from each mouse were prepared immediately after transcardial perfusion, with lysis buffer (50 mM Tris–HCl, pH 7.5, 150 mM NaCl, 1% Triton X-100, 0.1% SDS, 2 mM EDTA, 0.1 mM EGTA, 5 mM NaF, 1 mM Na_3_VO_4_, 5 mM Na_2_PO_4_) and 1× proteinase inhibitor cocktail (Halt^TM^, 78429, Thermofisher, Waltham, MA, USA).

### 4.2. Western Blot

#### 4.2.1. Protein Quantification

The standard BCA colorimetric method (Pierce BCA protein assay kit, 23225, Thermofisher, Waltham, MA, USA) was used for the determination of the total protein concentration of brain lysates. The brain lysates were diluted in a 1:100 ratio in a 96-well plate. The calibration curve was constructed with BSA standard with a final concentration of 15.625 at 1000 μg/mL, with 7 points serial dilution and using dH_2_O as a blank. Each point of the curve was performed in triplicate, obtaining R^2^ ≥ 0.99. In total, 100 μL of the diluted samples were mixed in a plate with 100 μL of working solution composed of commercial BCA solution and 4% m/V CuSO_4_ in a volume ratio of 50:1. The plate was incubated at 37 °C for 30 min on an orbital shaker at 50 RPM, until read on a Synergy HT^®^ plate reader at 562 nm.

#### 4.2.2. Western Blot Analysis

Brain samples (30 µg of protein) were prepared with loading buffer LDS NuPAGE (NP0008, Thermofisher, Waltham, MA, USA) and electrophoretically separated on 10% polyacrylamide gels and transferred to a nitrocellulose membrane. The membrane was blocked with 0.1% Tween 20 and 5% skim milk in Tris-buffered saline (TBS) for 1 h. Then, it was incubated with the primary antibody in the blocking buffer (used antibodies in Table 2). The primary antibodies were washed 10 min x 4, and the membranes were incubated with the secondary antibodies labelled with horseradish peroxidase according to the primary antibody species (Table 2).

The signal was detected through chemiluminescence with Westar Antares (CYANAGEN, XLS142,0250, Bologna, Italy) and scanned using a C-DiGit® Chemiluminescence Western Blot Scanner by LI-COR, according to the manufacturer’s instructions. Densitometry analysis was performed using the Li-cor system, with the Image Studio™ (version 6.1) for image capture. The background of the blot was subtracted to obtain consistent data. For analysis tools, ImageJ (version 1.54) was used, defining the region of interest (ROI) and drawing a rectangle around the band to fully enclose the entire signal for the largest band to be measured in that row. Measurement data were recorded and exported into an Excel spreadsheet. The same process was repeated for each protein of interest (40 kDa and 70 kDa CX3CL1 and CX3CR1) and the loading control. Formulas and calculations were performed according to ImageJ protocol, obtaining the final relative quantification values corresponding to the ratio of the net protein of interest band to the net loading control (β-tubulin), which was used to construct the graph. Complete Wester blots can be found in the Supplementary Materials.

### 4.3. RNA Isolation and Real-Time Reverse Transcription Quantitative Polymerase Chain Reaction (RT-qPCR) Assays

Total mRNA was extracted from the brain using TRIzol reagent (Invitrogen, Waltham, MA, USA) according to the manufacturer’s protocol. The purity of the RNA samples was assessed, including no-RT controls during the setup of the experiments. First-strand cDNA was made from the total RNA using M-MLV reverse transcriptase (Invitrogen, Waltham, MA, USA) with random primers. No-template controls (NTCs) were included with each set of RT-qPCR sample groups. The expression levels of CX3CL1, CX3CR1, and TGFβ were measured through real-time qPCR using SYBR Green I Master Mix Kit (Qiagen, Germantown, MD, USA). 

Primer sequences are shown in Table 3. Primer efficiency was assessed using a standard curve for each primer set. A regression coefficient was calculated, requiring values of R^2^ ≥ 0.99. The amplification efficiency was calculated using the slope of the regression line in the standard curve. For the Step One equipment used in these experiments, a slope close to −3.32 indicated a 100% PCR amplification efficiency. The amplification efficiency of our primers was between 95% and 105% of the slope specified for Step One.

Thermocycler conditions included an initial hold at 95 °C for 5 min, followed by a two-step PCR programme at 95 °C for 5 s and 60 °C for 10 s, repeated for 40 cycles in a Step One system (Applied Biosystems, Waltham, MA, USA). The amount of endogenous β-Actin mRNA was used as an internal control for qPCR in each sample.

Relative expression analysis by the ∆∆CT method, as described previously [97], was performed using the following formulas:

∆Ct = Ct (gene of interest) − Ct (housekeeping gene)

∆∆Ct = ∆Ct (treated/experimental sample) − ∆Ct (untreated sample)

$$RQ:2^{-∆∆CT}$$

After the RT-qPCR was performed, a melting analysis was performed for all the samples to identify the specificity of the product amplified during the PCR reaction. Melting curves can be found in the Supplementary Materials.

### 4.4. Statistical Analysis

Data were reported as means ± SE (SEM) for normally distributed data. The comparisons of mRNA expression (RT-qPCR) and protein level (Western blot) levels among the various experimental conditions and age groups were performed by using the Two-way mixed ANOVA, followed by Tukey’s test. The Fixed effects (type III) *p* value and F-test (DFn, DFd) were calculated and indicated in each figure legend. For simple comparisons between experimental conditions with a small number of independent experiments, a Kruskal–Wallis test, followed by the post hoc Dunn’s test, was used. All analyses were conducted with Graph Pad Software (version 10.6.1), including the preparation of the graphs for the figures. A *p* value < 0.05 was considered statistically significant.

## 5. Conclusions

- Ageing-related changes resulted in the highest levels of CX3CL1 and TGFβ in adults, with a significant decrease in old mice; CX3CR1 WB showed a similar effect, although there were no differences at the mRNA level.
- LPS administered acutely induced a conspicuous increase in CX3CL1 and CX3CR1 in adult mice, and the response in SRA^−/−^ mice was even more robust.
- TGFβ reduced the levels of CX3CL1, CX3CR1, and TGFβ induced by the acute treatment with LPS, although CX3CL1 mRNA was potentiated by TGFβ.
- Ageing effects on the TGFβ level can serve as a key regulator of neuroinflammation, being involved in augmented inflammatory activation.
- CX3CL1 serves regulatory functions in the activation of microglia and neuroinflammation. Modifications in membrane-associated and soluble CX3CL1 in ageing, as well as in CX3CR1, are interesting candidates for understanding ageing-associated changes in the neural regulation and neurotoxicity of microglia.

## Figures and Tables

**Table 1:** Mice used.

Mice	Age (m)	Number Mice
*Mus Musculus*/C57B6/–WT	3–7	65
*Mus Musculus*/C57B6/–WT	12–15	48
*Mus Musculus*/C57B6/–WT	>20	29
*Mus Musculus* /B6.Cg-Msr1tm1Csk/J/–SRA^−/−^	3–7	20
*Mus Musculus*/B6.Cg-Msr1tm1Csk/J/–SRA^−/−^	12–15	12
*Mus Musculus*/B6.Cg-Msr1tm1Csk/J/–SRA^−/−^	>20	12

Both male and female mice were included in similar proportions.

**Table 2:** Antibody information.

Antibody Name	Manufacturer Info	Concentration
α-CX3CL1, rabbit	14-7186-81 Invitrogen, Waltham, MA, USA	1:1000
α-CX3CR1 rabbit	14-6093-81, Invitrogen, Waltham, MA, USA	1:1000
α-β3 tubulin, mouse	SC-80005, Santa Cruz, Dallas, TX, USA	1:1000
Goat α-Rabbit IgG, H&L Chain	401315, Calbiochem, Darmstadt, Germany	1:5000
Goat α-Mouse IgG, H&L Chain	401215, Calbiochem, Darmstadt, Germany	1:10,000

**Table 3:** Primers sequences.

Primer	Sequence	Amplification Product Length
CX3CL1-fw	AACCAGTTGTAGGCCTGAGC	129 bp
CX3CL1-rev	CACATTCTGCTCTGGGAGGG	
CX3CR1-fw	CCCCTTTATCTACGCCTTTGC	180 bp
CX3CR1-rev	CCATCTCCCTCGCTTGTGT	
TGFβ-fw	CTATGCTAAAGAGGTCACCCG	123 bp
TGFβ-rev	ACTGCTTCCCGAATGTCTG	
β-actin-fw	GATGACCCAGATCATGTTTG	292 bp
β-actin-rev	CTTCTCTTTGATGTCACGCA	

## Data Availability

The raw data supporting the conclusions of this article will be made available by the authors on request. The Materials, data, and protocols associated with this publication are available to the academic community from the corresponding authors upon reasonable request. Please write to the corresponding authors to provide the materials or information that are required.

## Supplementary Materials

The following supporting information can be downloaded at: https://www.mdpi.com/article/10.3390/ijms262311378/s1.

## Abbreviations

The following abbreviations are used in this manuscript:

AAV	Adeno-associated virus
Aβ	β amyloid
aCSF	Artificial cerebrospinal fluid
AD	Alzheimer’s disease
ALS	Amyotrophic lateral sclerosis
BBB	Blood–brain barrier
β-Tub	Beta-tubulin
CNS	Central nervous system
CX3CL1	Fractalkine
CX3CR1	Fractalkine receptor
ERK	Extracellular signal-regulated kinase
ICV	Intracerebroventricular
IFNγ	Interferon γ
IL1β	Interleukin 1β
IL6	Interleukin 6
IL10	Interleukin 10
i.p.	Intra peritoneal
i.t.	Intrathecal
LPS	Lipopolysaccharide
MAPKs	Mitogen-activated protein kinases
MCP1	Monocyte chemoattractant protein-1
mCX3CL1	Membrane-bound fractalkine
MKP-1	MAP kinase fosfatase-1
MPTP	1-methyl-4-phenyl-1,2,3,6-tetrahydropyridine
mRNA	Messenger ribonucleic acid
NO	Nitric oxide
Nrf2	Nuclear factor erythroid related factor 2
PBS	Phosphate-buffered saline
PD	Parkinson’s disease
PI3K	Phosphatidylinositol 3-kinase
rAAV	Recombinant adeno-associated virus
ROS	Reactive oxygen species
RT	Reverse transcriptase
RT-qPCR	Reverse Transcription Quantitative Polymerase Chain Reaction
sCX3CL1	Soluble fractalkine
SN	Substantia nigra
SNpc	Substantia nigra pars compacta
SPARC	Secreted protein acidic and rich in cysteine
SRA	Scavenger receptor type A
SRA^−/−^	Scavenger receptor type A knockout
TGFβ	Transforming growth factor β
TNFα	Tumour necrosis factor α
WT	Wild type
