# Supplementary material for: Age-Related Changes in Neuron–Microglia Interaction Mediated by Fractalkine Under Inflammatory Conditions
Source: Int J Mol Sci. 2025 Nov 25;26(23):11378. doi: 10.3390/ijms262311378 (PMC12692065; doi:10.3390/ijms262311378)
Supplement: Supplementary file 1 [file ijms-26-11378-s001.zip › SupMat-MeltingCurvesRT-qPCR-Final.pdf]

Age-related changes in neuron-microglia interaction mediated by Fractalkine under inflammatory conditions

Melting Curves: Melting curve analysis was used to evaluate the specificity of the amplified products. The graphs show a single peak for the amplified product of each primer, indicating its specificity. When observed, additional peaks are at temperatures below 80 C, indicating they are primer dimers. When more than one curve appeared on the melting curves, all samples of the qRT-PCR product were runed in agarose gels to assess if additional bands were visualized. No additional bands were obtained suggesting that the amplification at low temperature visualized in the melting curve was very limited and considered non-detectable. In samples where more than one peak was observed, the result was discarded and the sample tested again.

Melting curve for β-actin:

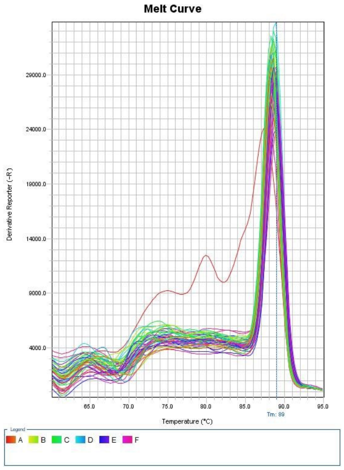

β-actin samples 1-23

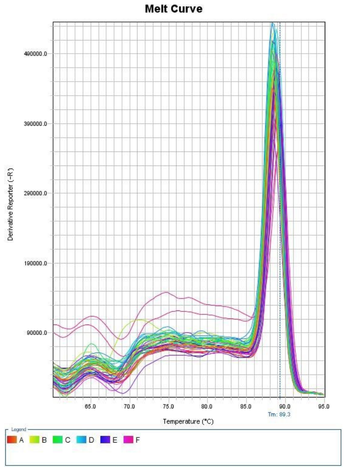

β-actin samples 24-46

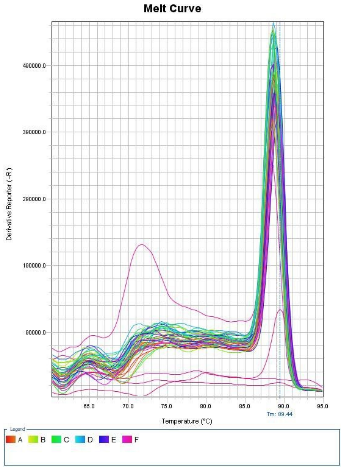

β-actin samples 47-68

Melting curve for CX3CL1

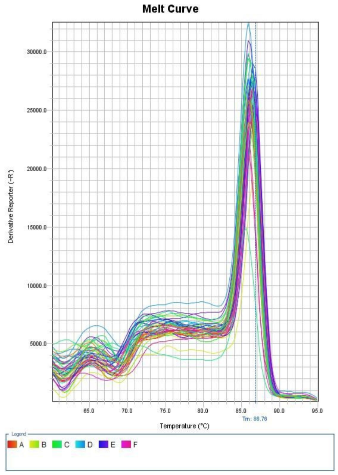

CX3CL1 samples 1-23

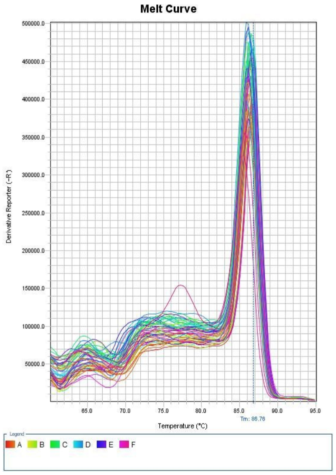

CX3CL1 samples 24-46

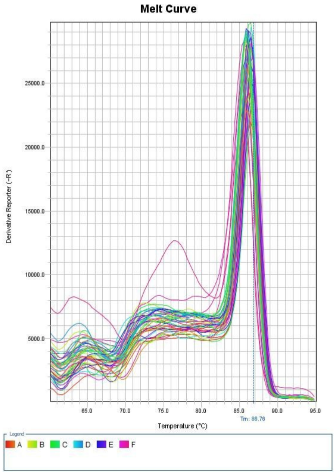

CX3CL1 samples 47-6

Melting curve for CX3CR1:

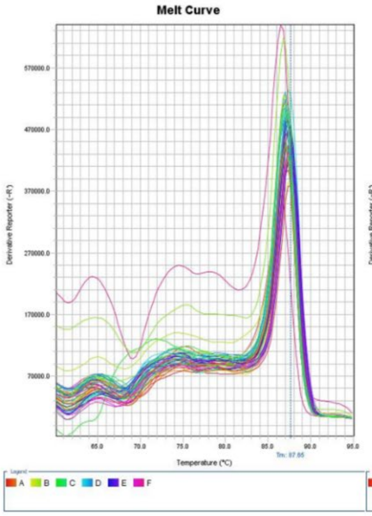

CX3CR1 samples 1-23

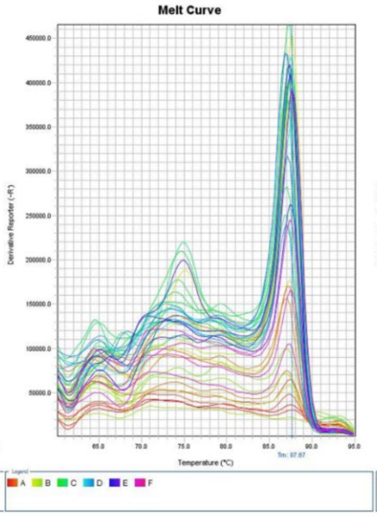

CX3CR1 samples 24-46

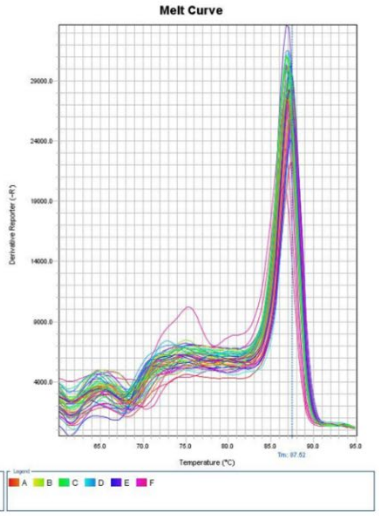

CX3CR1 samples 47-68

Melting curve for TGFβ:

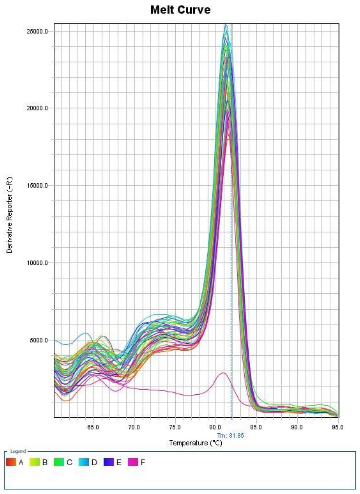

TGFβ samples 1-23

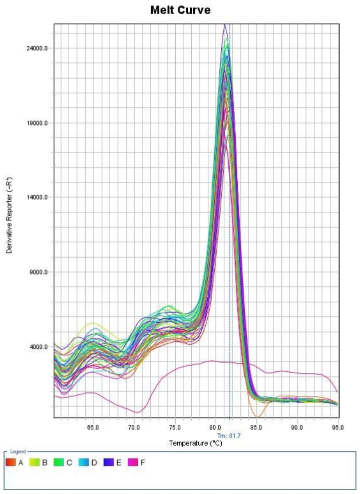

TGFβ samples 24-46

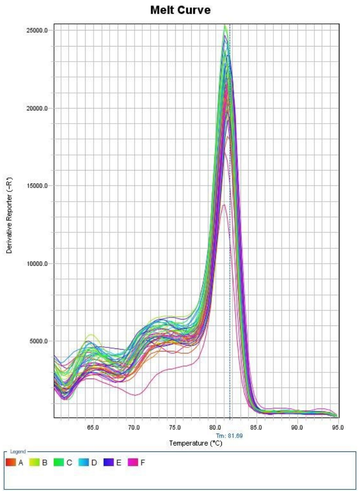

TGFβ samples 47-68
