# Supplementary material for: Age-Related Changes in Neuron–Microglia Interaction Mediated by Fractalkine Under Inflammatory Conditions
Source: Int J Mol Sci. 2025 Nov 25;26(23):11378. doi: 10.3390/ijms262311378 (PMC12692065; doi:10.3390/ijms262311378)
Supplement: Supplementary file 1 [file ijms-26-11378-s001.zip › SupMat-WBsPaperFKN-Final.pdf]

Age-related changes in neuron-microglia interaction mediated by Fractalkine under inflammatory conditions

Western blots

Fig 2 & 4

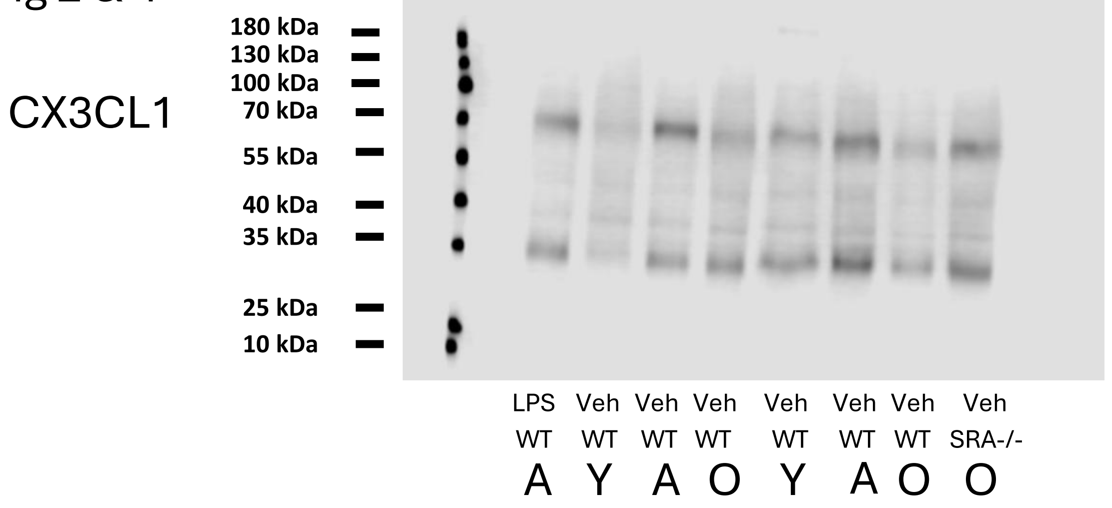

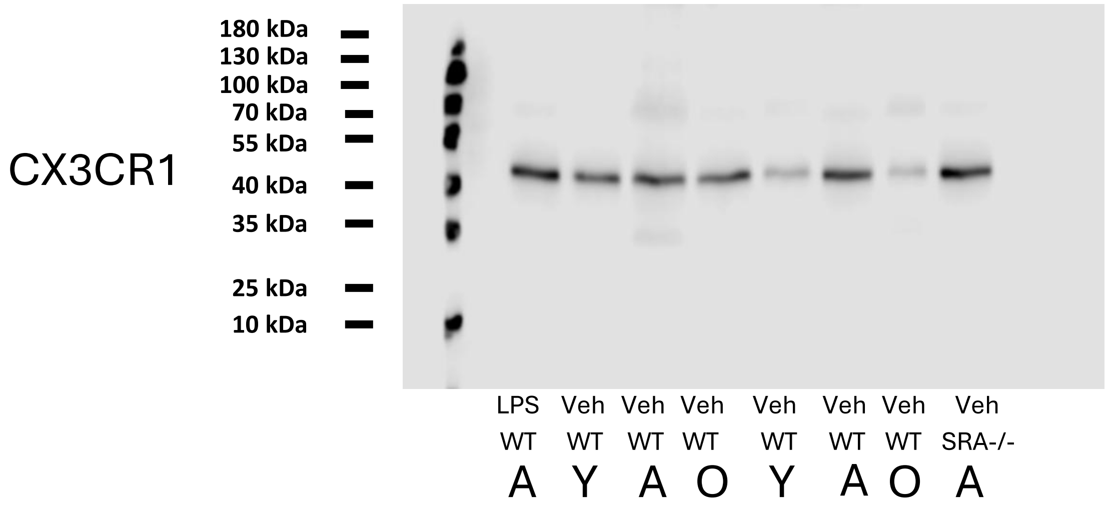

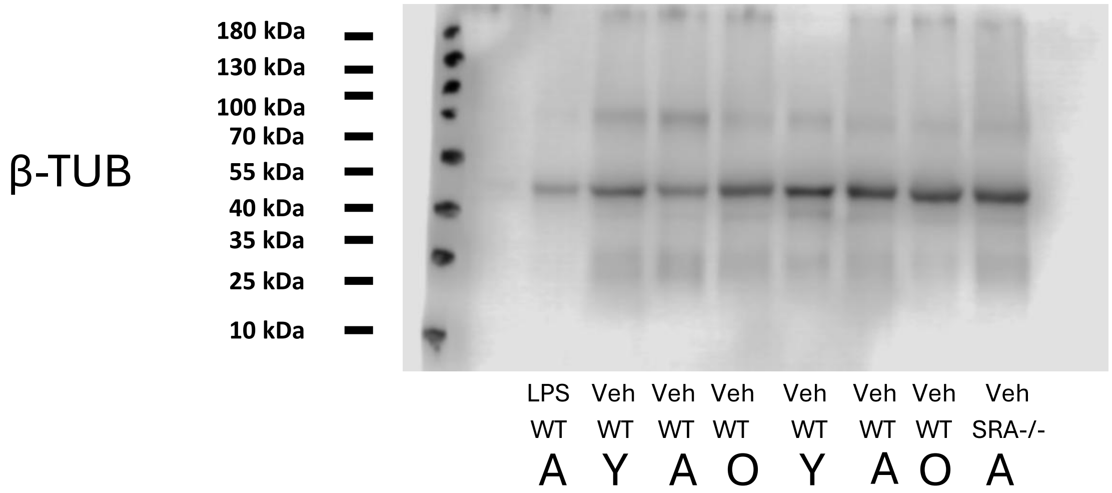

Fig 2 & 4

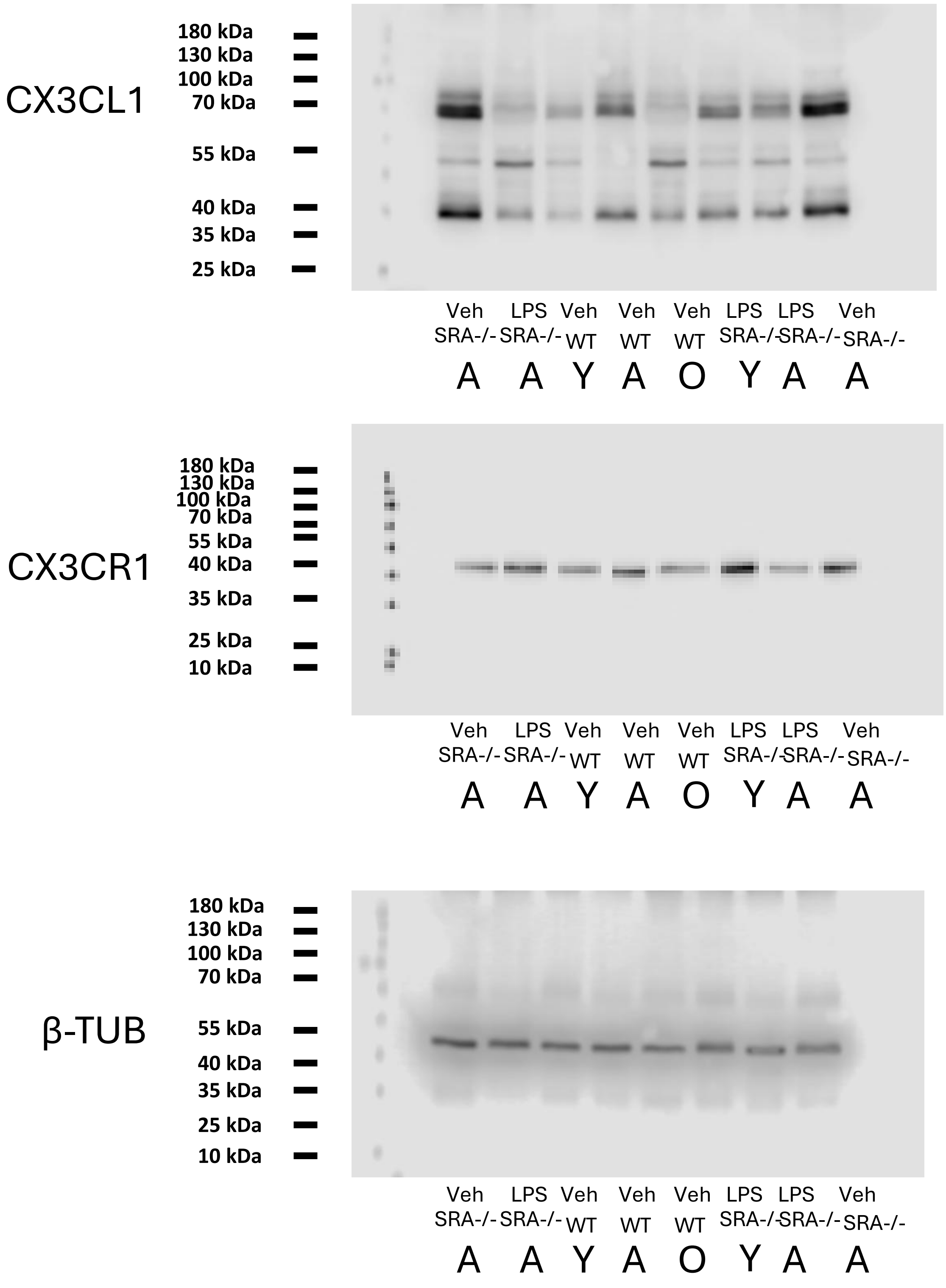
CX3CL1
180 kDa
130 kDa
100 kDa
70 kDa
55 kDa
40 kDa
35 kDa
25 kDa
Veh SRA-/- LPS SRA-/- Veh WT Veh WT Veh WT LPS SRA-/- LPS SRA-/- Veh SRA-/-
A A Y A O Y A A
CX3CR1
180 kDa
130 kDa
100 kDa
70 kDa
55 kDa
40 kDa
35 kDa
25 kDa
10 kDa
Veh SRA-/- LPS SRA-/- Veh WT Veh WT Veh WT LPS SRA-/- LPS SRA-/- Veh SRA-/-
A A Y A O Y A A
β-TUB
180 kDa
130 kDa
100 kDa
70 kDa
55 kDa
40 kDa
35 kDa
25 kDa
10 kDa
Veh SRA-/- LPS SRA-/- Veh WT Veh WT Veh WT LPS SRA-/- LPS SRA-/- Veh SRA-/-
A A Y A O Y A A

Fig 2 & 4 – CX3CR1

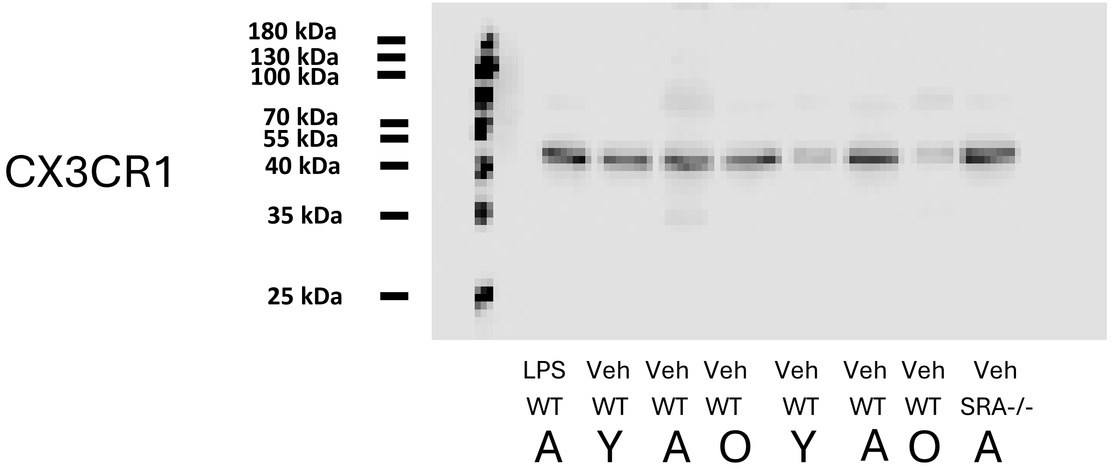

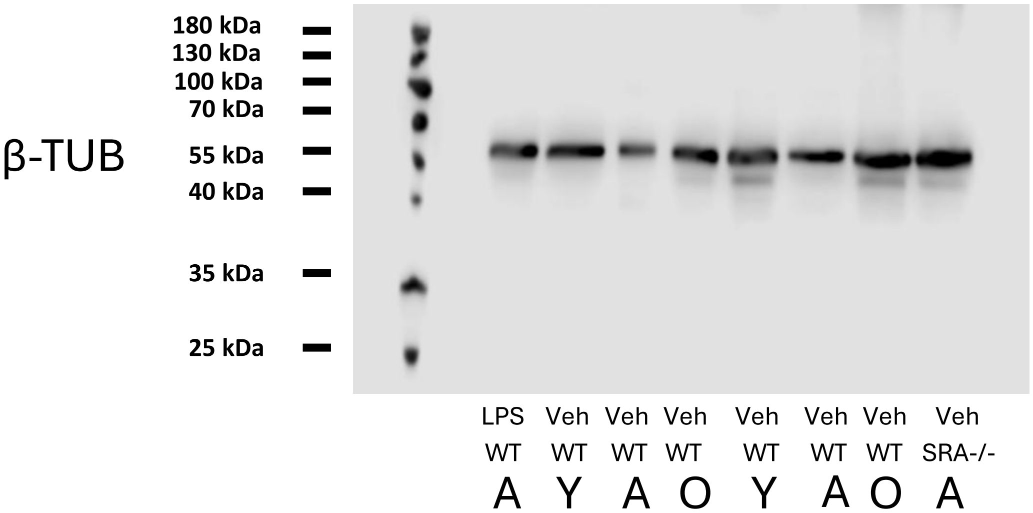

Fig 2 & 4

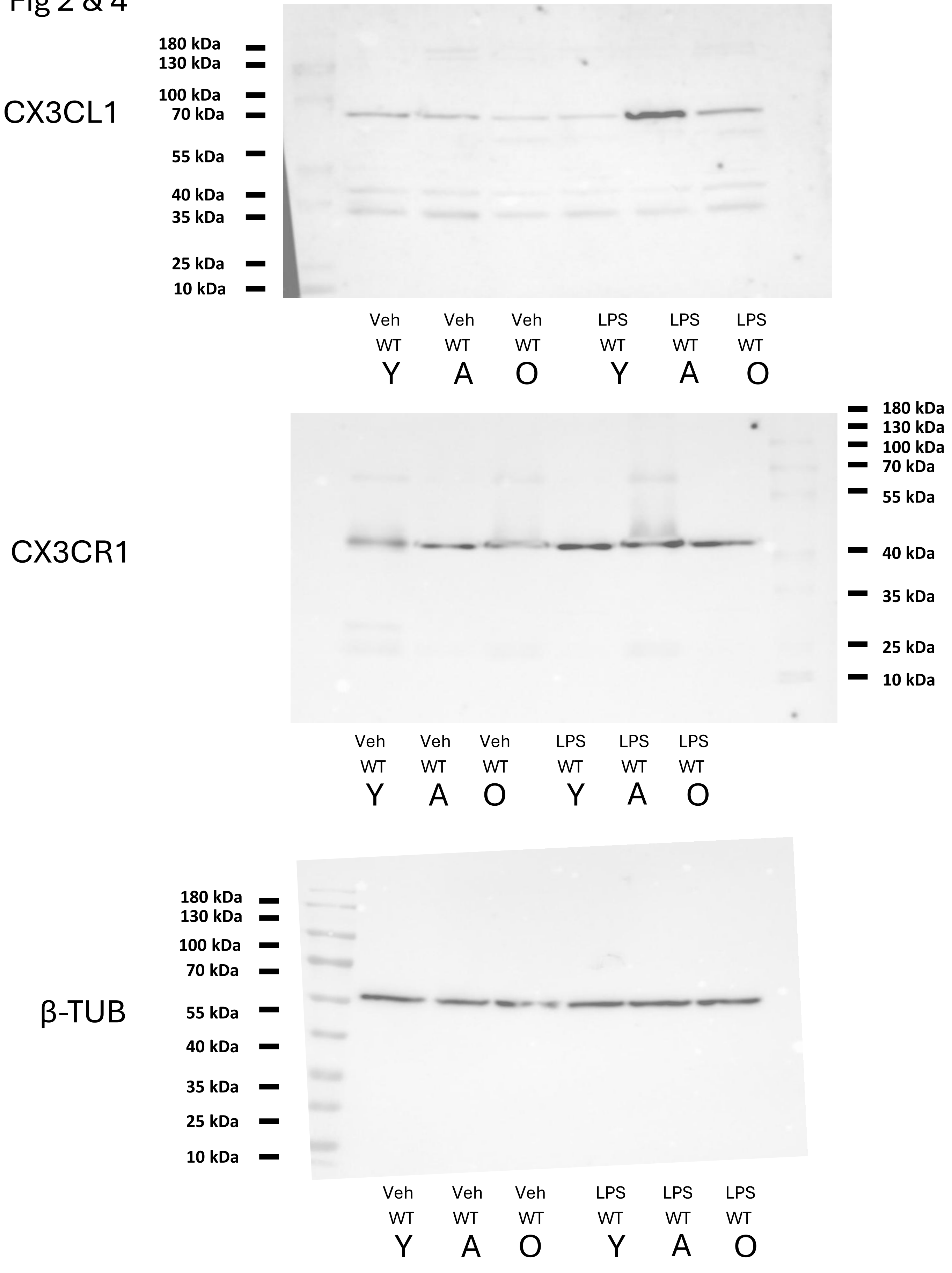
CX3CL1
180 kDa
130 kDa
100 kDa
70 kDa
55 kDa
40 kDa
35 kDa
25 kDa
10 kDa
Veh WT Y
Veh WT A
Veh WT O
LPS WT Y
LPS WT A
LPS WT O
CX3CR1
180 kDa
130 kDa
100 kDa
70 kDa
55 kDa
40 kDa
35 kDa
25 kDa
10 kDa
Veh WT Y
Veh WT A
Veh WT O
LPS WT Y
LPS WT A
LPS WT O
β-TUB
180 kDa
130 kDa
100 kDa
70 kDa
55 kDa
40 kDa
35 kDa
25 kDa
10 kDa
Veh WT Y
Veh WT A
Veh WT O
LPS WT Y
LPS WT A
LPS WT O

Fig 2 & 4

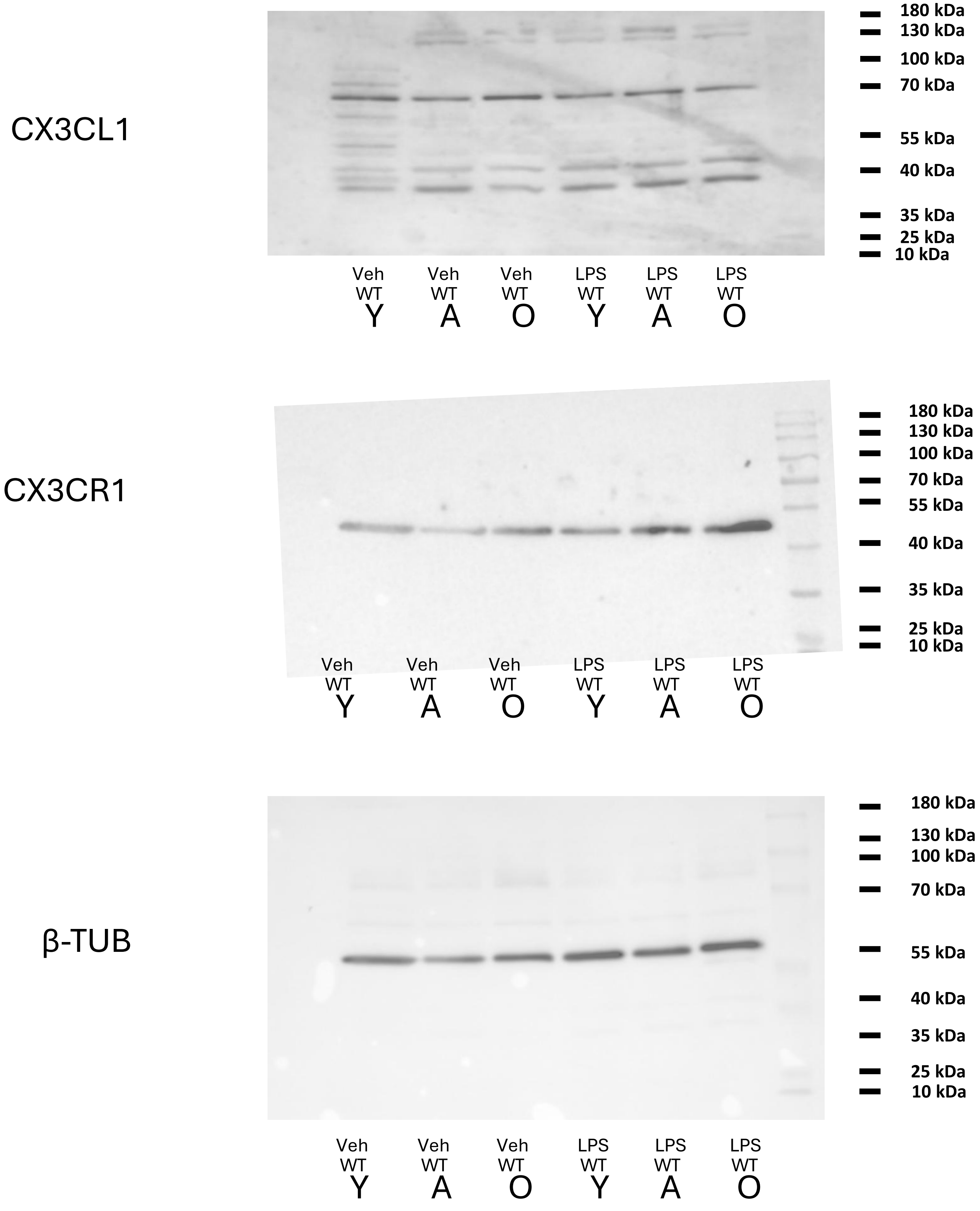
CX3CL1
180 kDa
130 kDa
100 kDa
70 kDa
55 kDa
40 kDa
35 kDa
25 kDa
10 kDa
Veh WT Y
Veh WT A
Veh WT O
LPS WT Y
LPS WT A
LPS WT O
CX3CR1
180 kDa
130 kDa
100 kDa
70 kDa
55 kDa
40 kDa
35 kDa
25 kDa
10 kDa
Veh WT Y
Veh WT A
Veh WT O
LPS WT Y
LPS WT A
LPS WT O
β-TUB
180 kDa
130 kDa
100 kDa
70 kDa
55 kDa
40 kDa
35 kDa
25 kDa
10 kDa
Veh WT Y
Veh WT A
Veh WT O
LPS WT Y
LPS WT A
LPS WT O

Fig 4

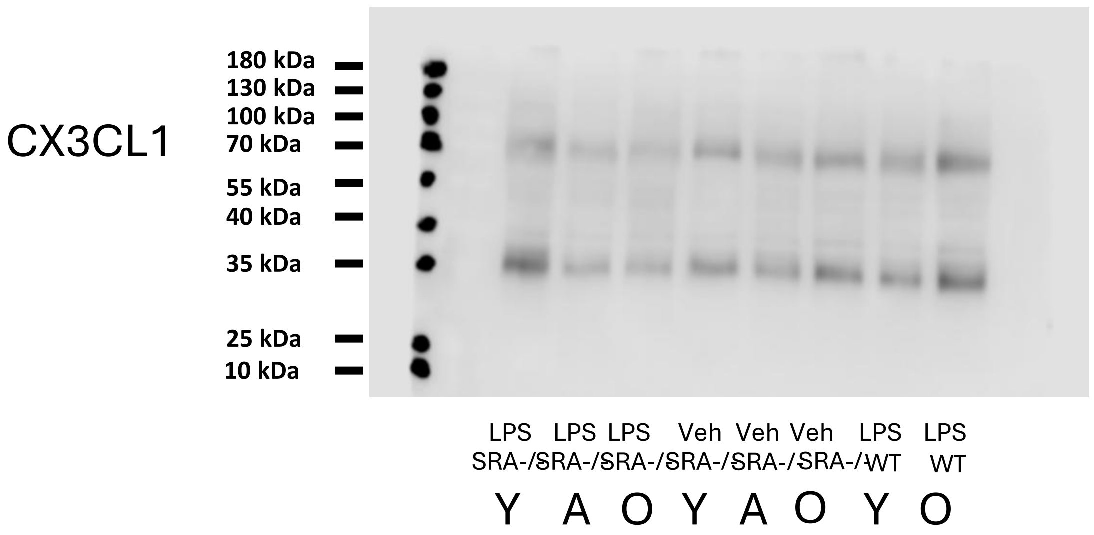
CX3CL1
180 kDa
130 kDa
100 kDa
70 kDa
55 kDa
40 kDa
35 kDa
25 kDa
10 kDa
LPS SRA-/-
LPS SRA-/-
LPS SRA-/-
Veh SRA-/-
Veh SRA-/-
Veh SRA-/-
LPS WT
LPS WT
Y A O Y A O Y O

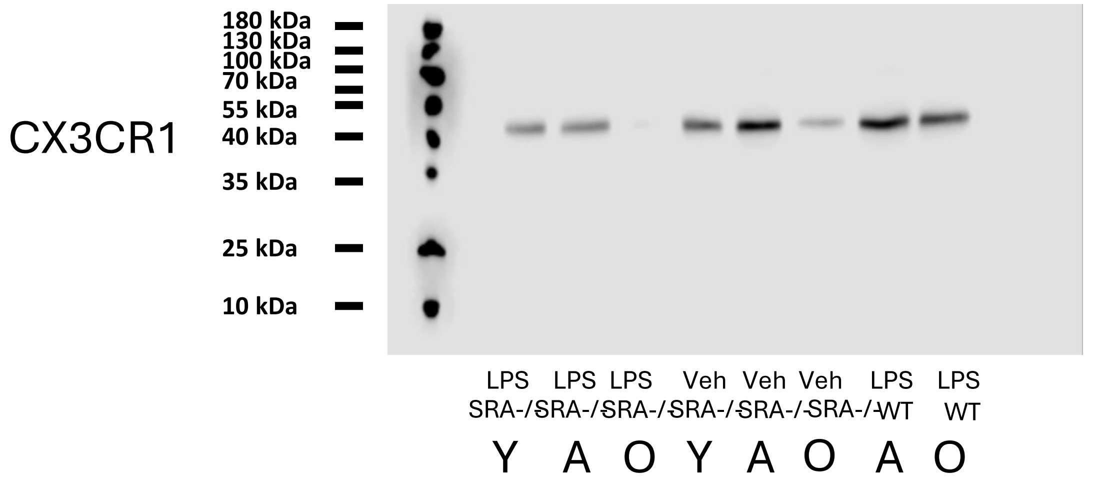
CX3CR1
180 kDa
130 kDa
100 kDa
70 kDa
55 kDa
40 kDa
35 kDa
25 kDa
10 kDa
LPS SRA-/-
LPS SRA-/-
LPS SRA-/-
Veh SRA-/-
Veh SRA-/-
Veh SRA-/-
LPS WT
LPS WT
Y A O Y A O A O

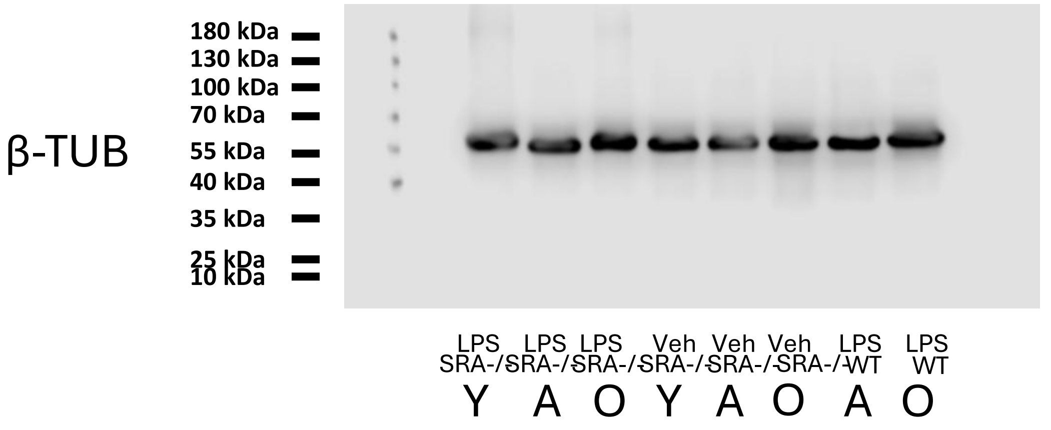
β-TUB
180 kDa
130 kDa
100 kDa
70 kDa
55 kDa
40 kDa
35 kDa
25 kDa
10 kDa
LPS SRA-/-
LPS SRA-/-
LPS SRA-/-
Veh SRA-/-
Veh SRA-/-
Veh SRA-/-
LPS WT
LPS WT
Y A O Y A O A O

Fig 4

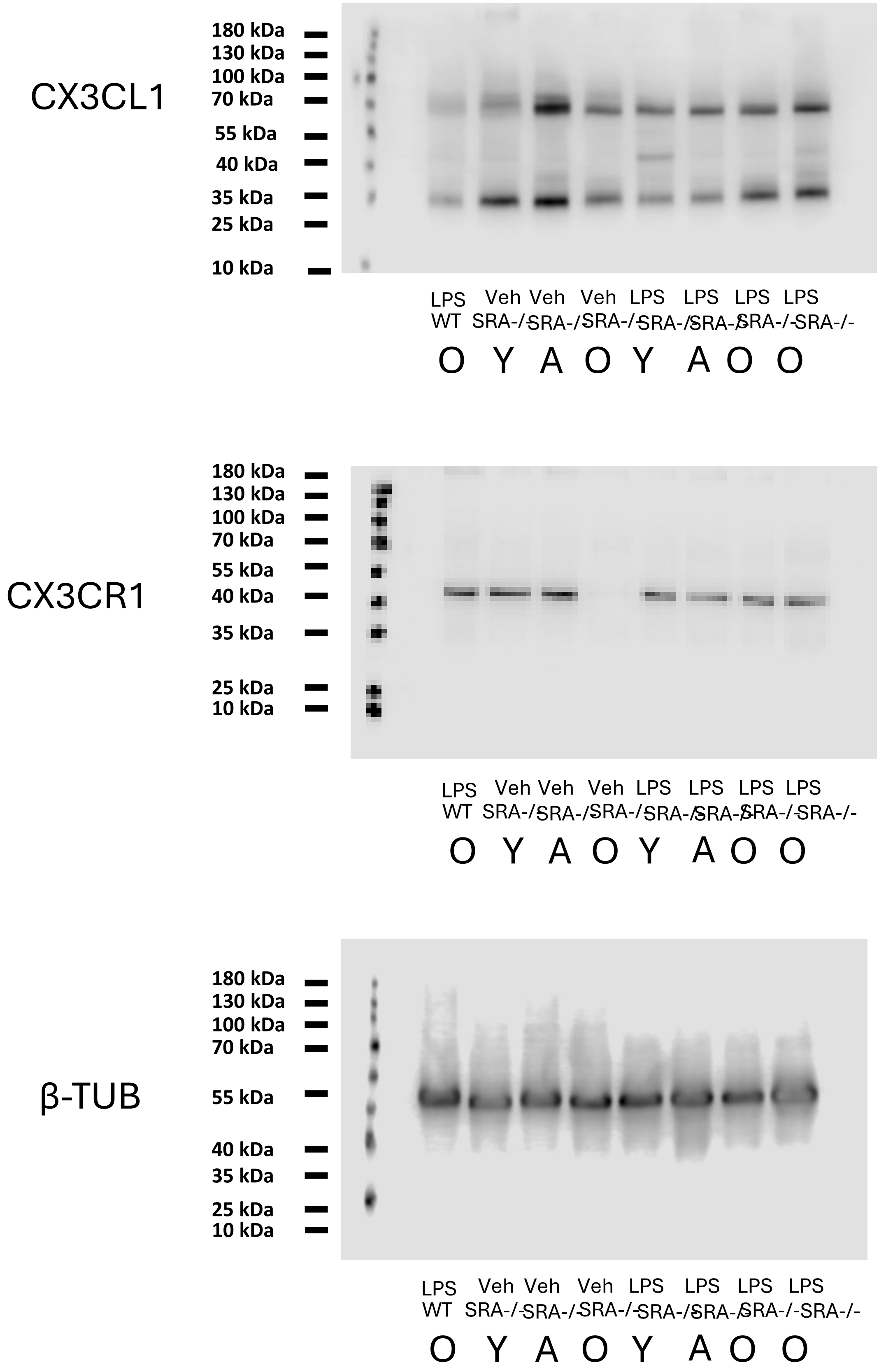
CX3CL1
180 kDa
130 kDa
100 kDa
70 kDa
55 kDa
40 kDa
35 kDa
25 kDa
10 kDa
LPS WT
Veh SRA-/-
Veh SRA-/-
Veh SRA-/-
LPS SRA-/-
LPS SRA-/-
LPS SRA-/-
LPS SRA-/-
O Y A O Y A O O
CX3CR1
180 kDa
130 kDa
100 kDa
70 kDa
55 kDa
40 kDa
35 kDa
25 kDa
10 kDa
LPS WT
Veh SRA-/-
Veh SRA-/-
Veh SRA-/-
LPS SRA-/-
LPS SRA-/-
LPS SRA-/-
LPS SRA-/-
O Y A O Y A O O
β-TUB
180 kDa
130 kDa
100 kDa
70 kDa
55 kDa
40 kDa
35 kDa
25 kDa
10 kDa
LPS WT
Veh SRA-/-
Veh SRA-/-
Veh SRA-/-
LPS SRA-/-
LPS SRA-/-
LPS SRA-/-
LPS SRA-/-
O Y A O Y A O O

Fig 2 & 4

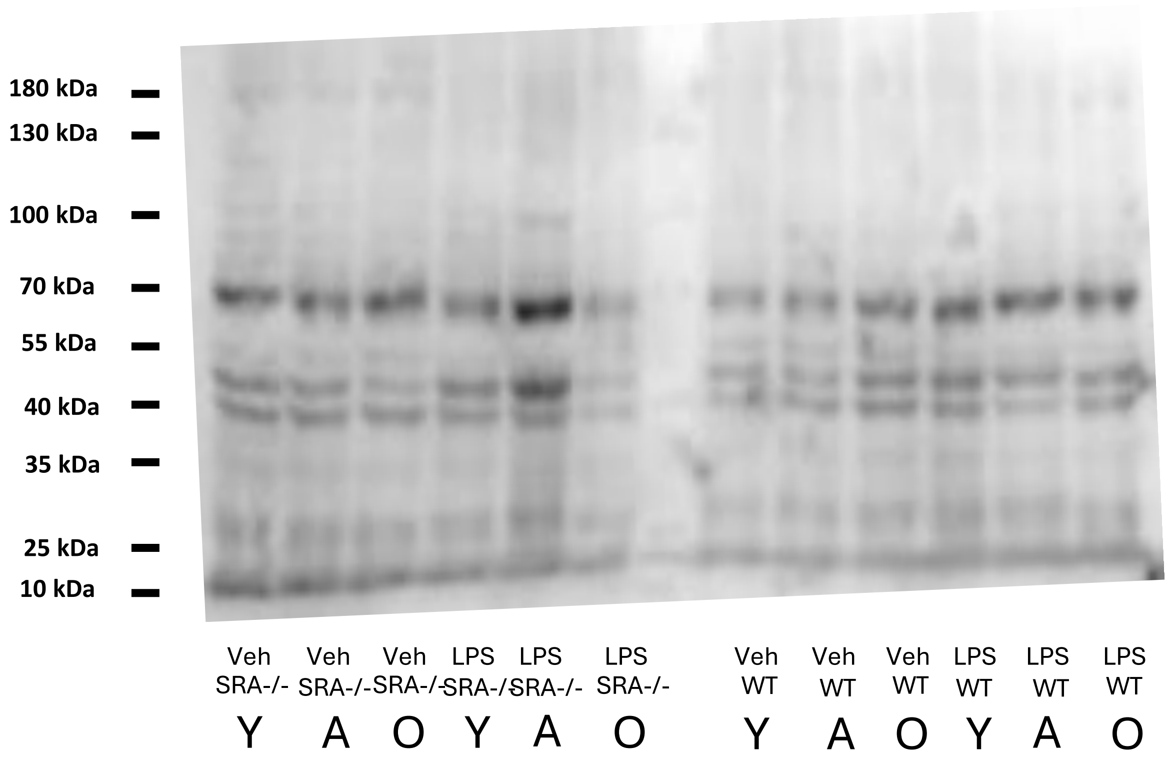
180 kDa
130 kDa
100 kDa
70 kDa
55 kDa
40 kDa
35 kDa
25 kDa
10 kDa
Veh SRA-/- Y
Veh SRA-/- A
Veh SRA-/- O
LPS SRA-/- Y
LPS SRA-/- A
LPS SRA-/- O
Veh WT Y
Veh WT A
Veh WT O
LPS WT Y
LPS WT A
LPS WT O

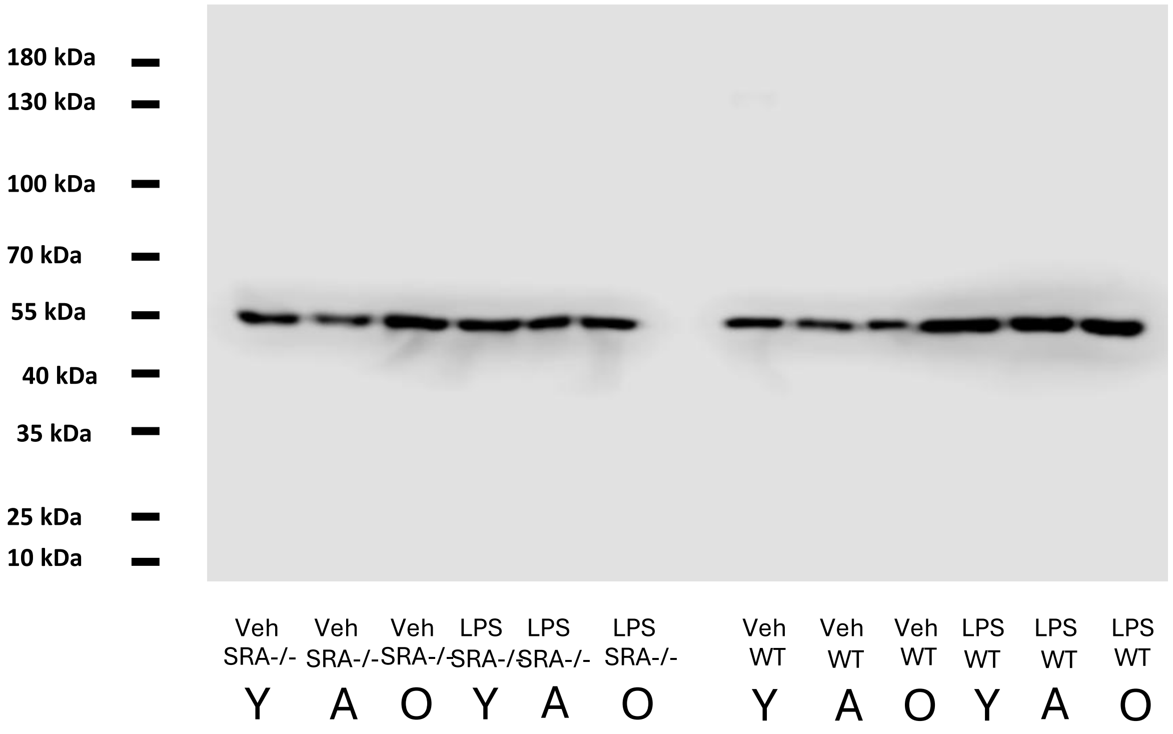
180 kDa
130 kDa
100 kDa
70 kDa
55 kDa
40 kDa
35 kDa
25 kDa
10 kDa
Veh SRA-/- Y
Veh SRA-/- A
Veh SRA-/- O
LPS SRA-/- Y
LPS SRA-/- A
LPS SRA-/- O
Veh WT Y
Veh WT A
Veh WT O
LPS WT Y
LPS WT A
LPS WT O

Fig 2 & 4 – CX3CR1

CX3CR1

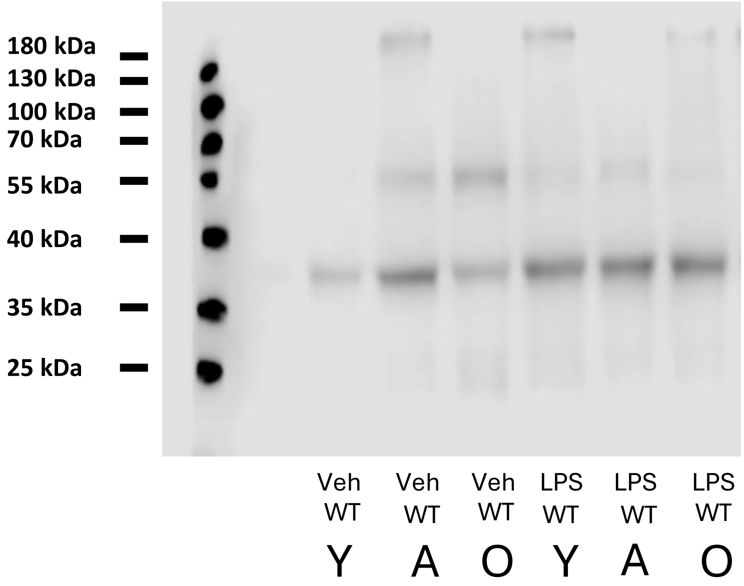

β-TUB

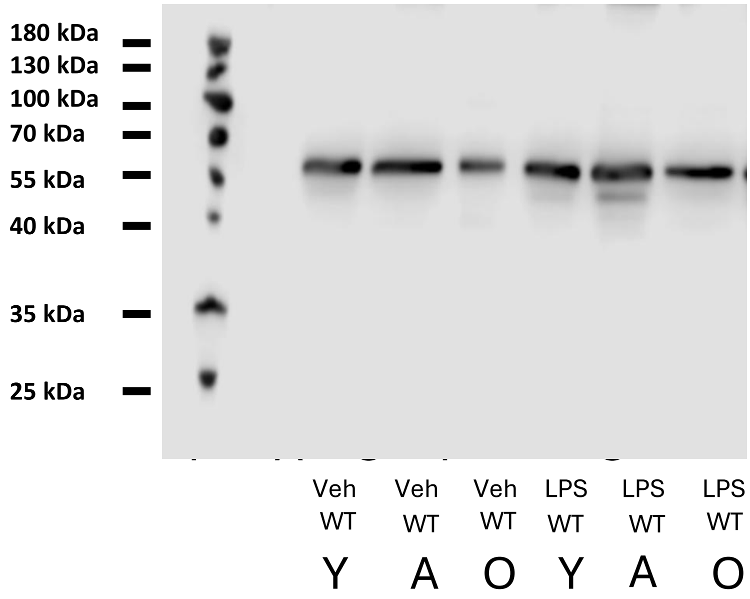

Fig 2 & 4 – CX3CR1

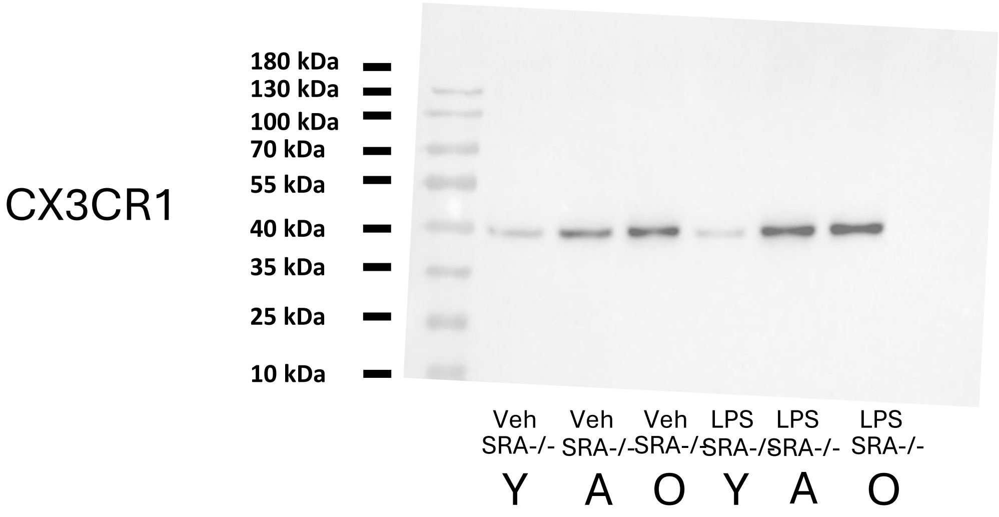

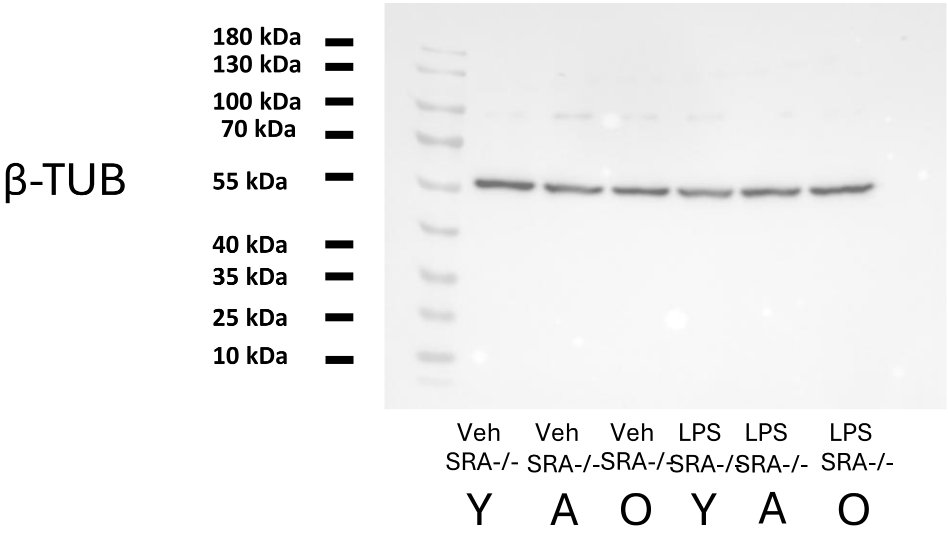

Fig 4

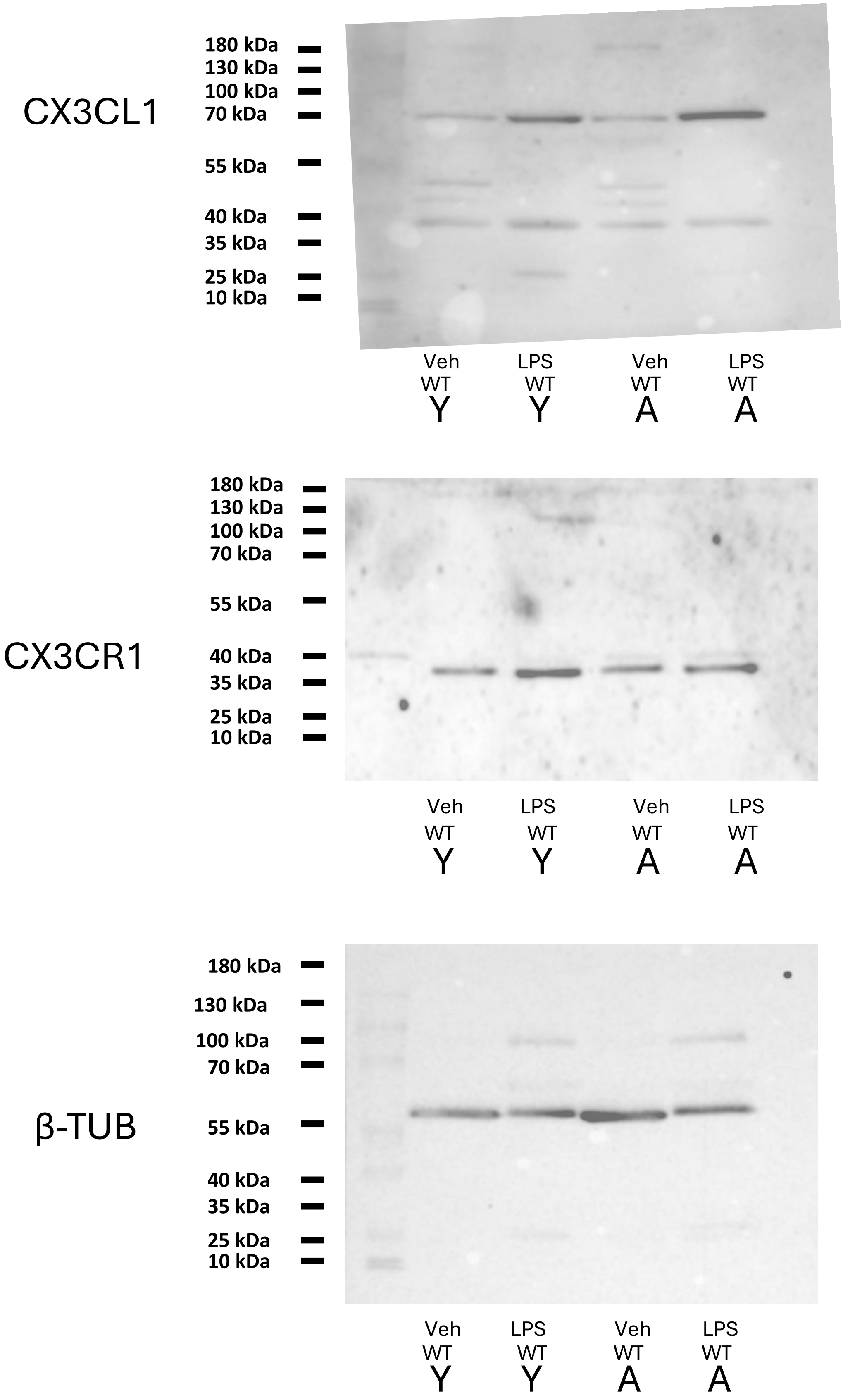
CX3CL1
180 kDa
130 kDa
100 kDa
70 kDa
55 kDa
40 kDa
35 kDa
25 kDa
10 kDa
Veh WT Y
LPS WT Y
Veh WT A
LPS WT A
CX3CR1
180 kDa
130 kDa
100 kDa
70 kDa
55 kDa
40 kDa
35 kDa
25 kDa
10 kDa
Veh WT Y
LPS WT Y
Veh WT A
LPS WT A
β-TUB
180 kDa
130 kDa
100 kDa
70 kDa
55 kDa
40 kDa
35 kDa
25 kDa
10 kDa
Veh WT Y
LPS WT Y
Veh WT A
LPS WT A

Fig 4

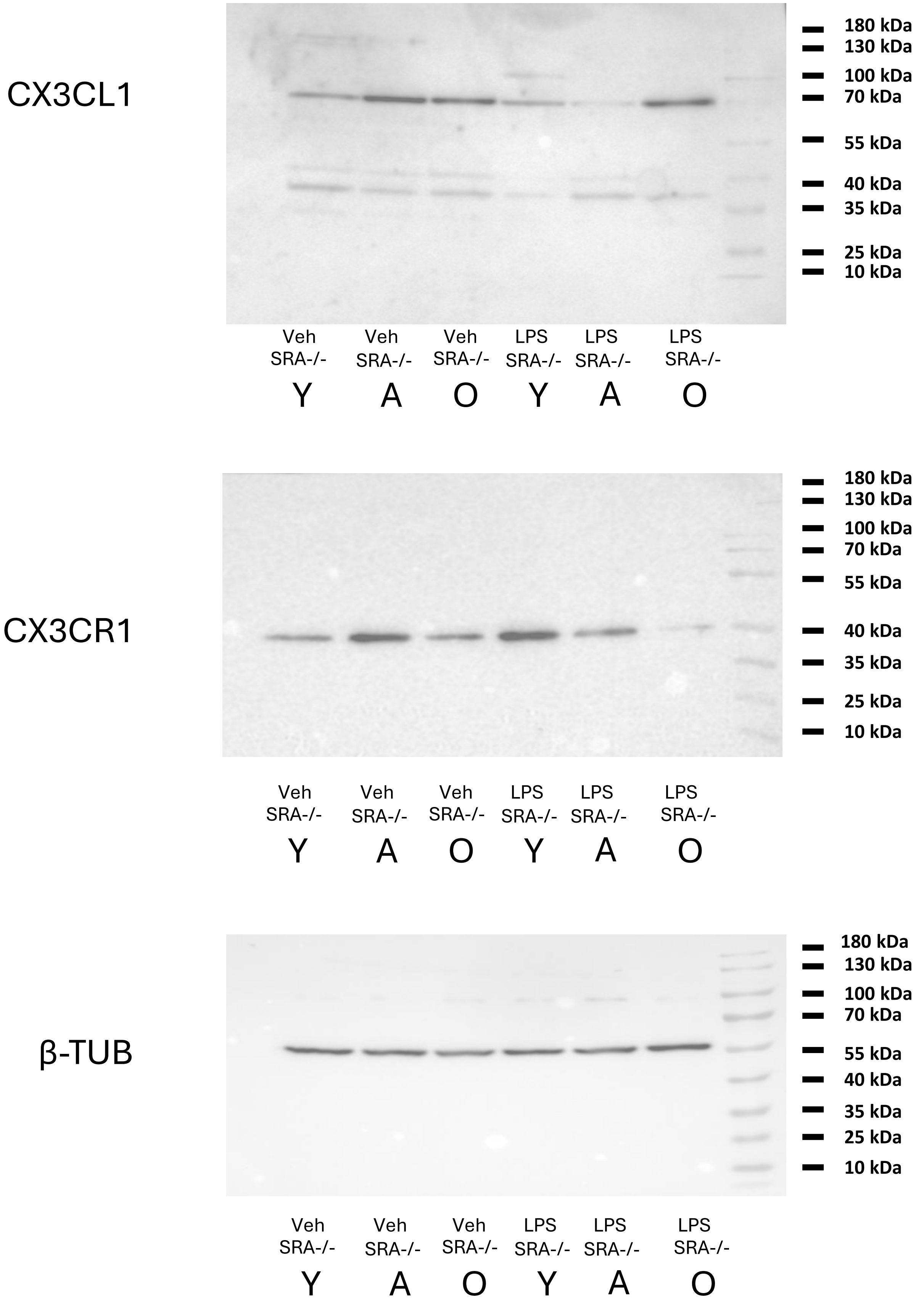
CX3CL1
180 kDa
130 kDa
100 kDa
70 kDa
55 kDa
40 kDa
35 kDa
25 kDa
10 kDa
Veh SRA-/- Y
Veh SRA-/- A
Veh SRA-/- O
LPS SRA-/- Y
LPS SRA-/- A
LPS SRA-/- O
CX3CR1
180 kDa
130 kDa
100 kDa
70 kDa
55 kDa
40 kDa
35 kDa
25 kDa
10 kDa
Veh SRA-/- Y
Veh SRA-/- A
Veh SRA-/- O
LPS SRA-/- Y
LPS SRA-/- A
LPS SRA-/- O
β-TUB
180 kDa
130 kDa
100 kDa
70 kDa
55 kDa
40 kDa
35 kDa
25 kDa
10 kDa
Veh SRA-/- Y
Veh SRA-/- A
Veh SRA-/- O
LPS SRA-/- Y
LPS SRA-/- A
LPS SRA-/- O

Fig 2 & 4

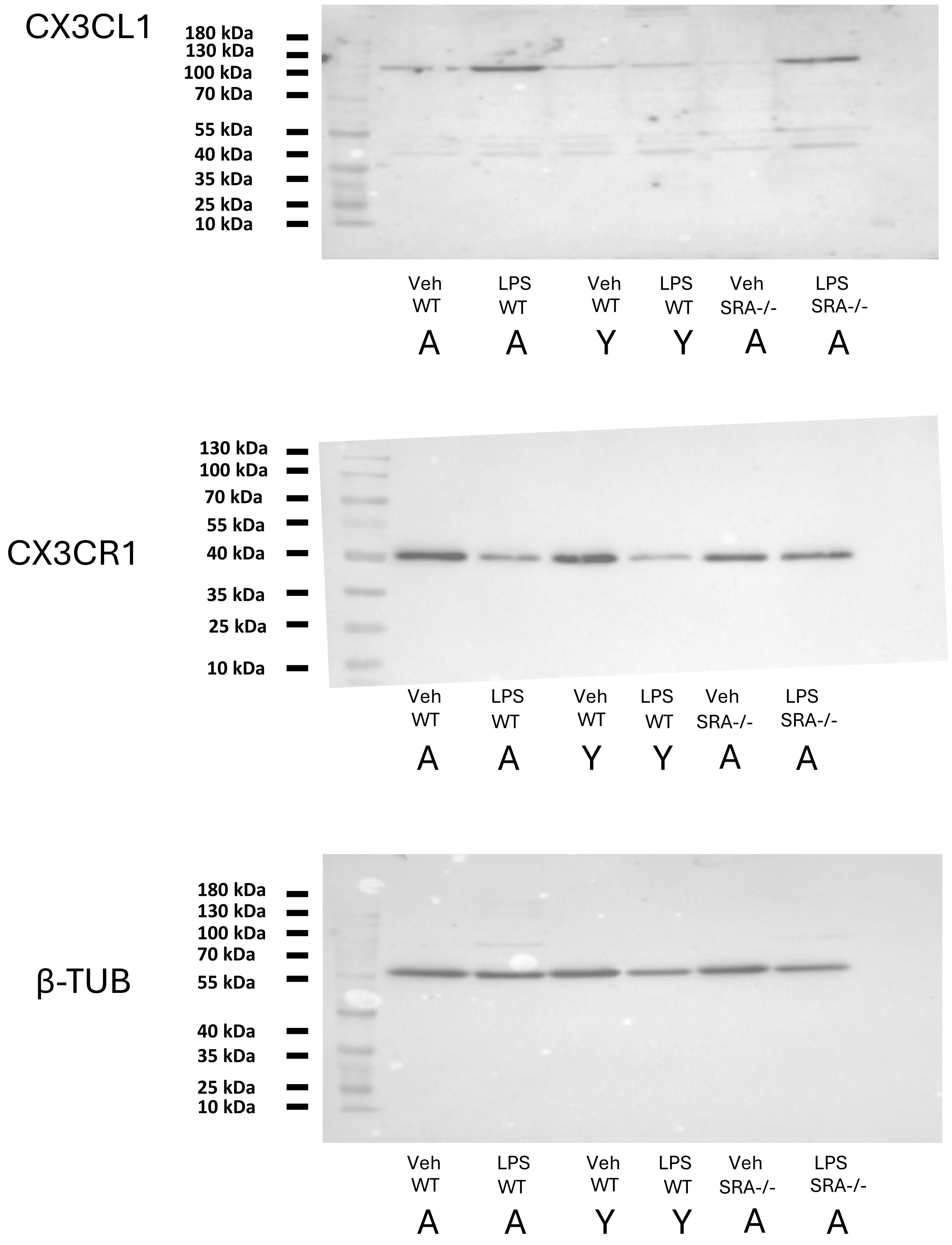
CX3CL1
180 kDa
130 kDa
100 kDa
70 kDa
55 kDa
40 kDa
35 kDa
25 kDa
10 kDa
Veh WT
LPS WT
Veh WT
LPS WT
Veh SRA-/-
LPS SRA-/-
A A Y Y A A
CX3CR1
130 kDa
100 kDa
70 kDa
55 kDa
40 kDa
35 kDa
25 kDa
10 kDa
Veh WT
LPS WT
Veh WT
LPS WT
Veh SRA-/-
LPS SRA-/-
A A Y Y A A
β-TUB
180 kDa
130 kDa
100 kDa
70 kDa
55 kDa
40 kDa
35 kDa
25 kDa
10 kDa
Veh WT
LPS WT
Veh WT
LPS WT
Veh SRA-/-
LPS SRA-/-
A A Y Y A A

Fig 4 -- CX3CR1

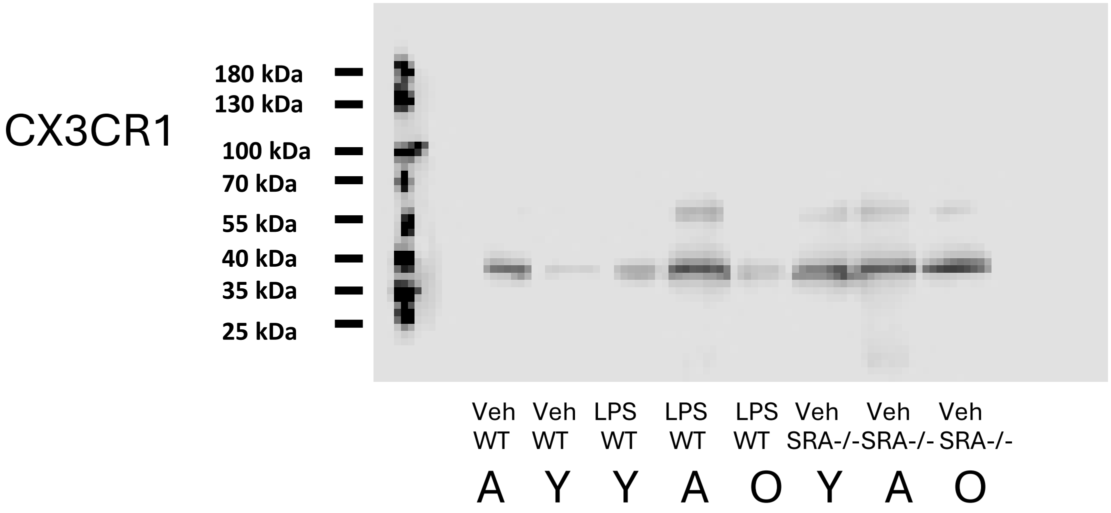
CX3CR1
180 kDa
130 kDa
100 kDa
70 kDa
55 kDa
40 kDa
35 kDa
25 kDa
Veh WT
Veh WT
LPS WT
LPS WT
LPS WT
Veh SRA-/-
Veh SRA-/-
Veh SRA-/-
A Y Y A O Y A O

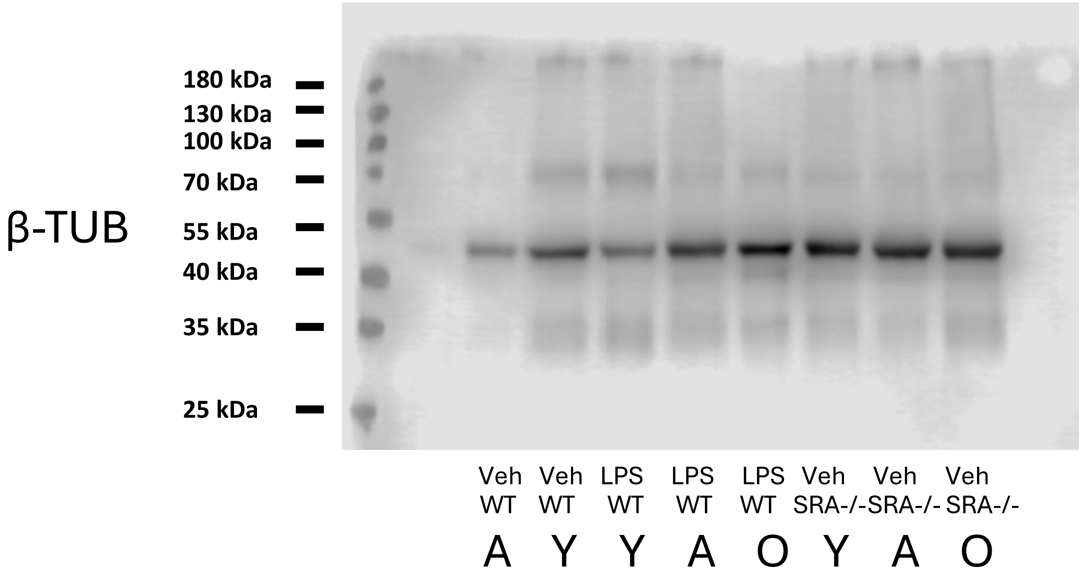
β-TUB
180 kDa
130 kDa
100 kDa
70 kDa
55 kDa
40 kDa
35 kDa
25 kDa
Veh WT
Veh WT
LPS WT
LPS WT
LPS WT
Veh SRA-/-
Veh SRA-/-
Veh SRA-/-
A Y Y A O Y A O

Fig 4 – CX3CR1

CX3CR1

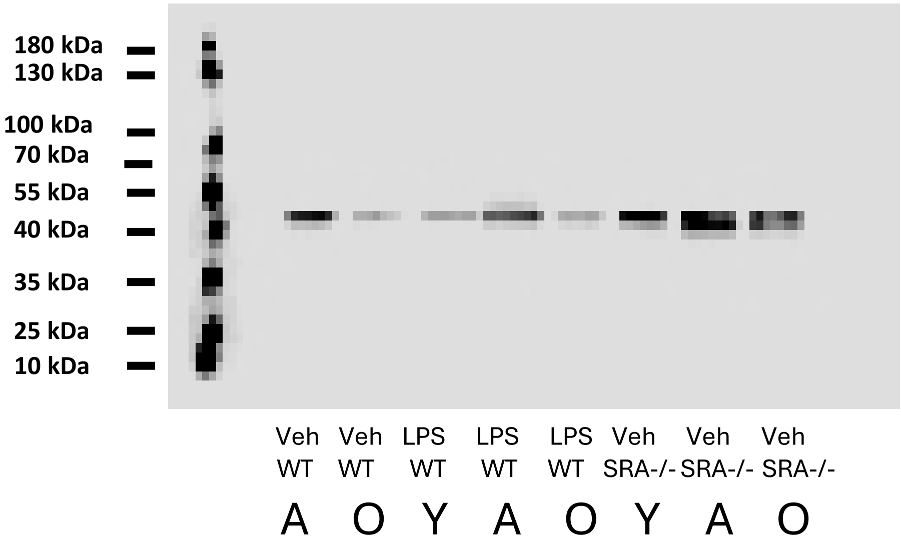

β-TUB

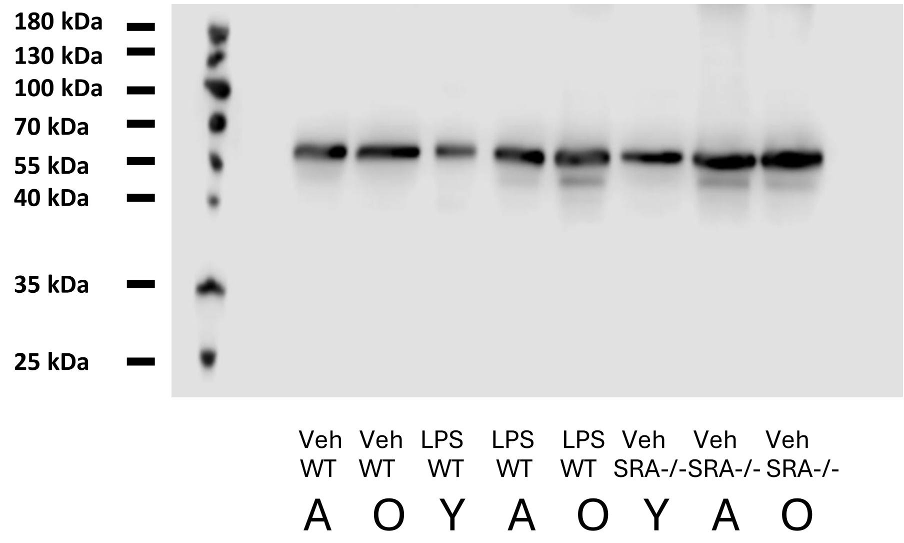

Fig 5

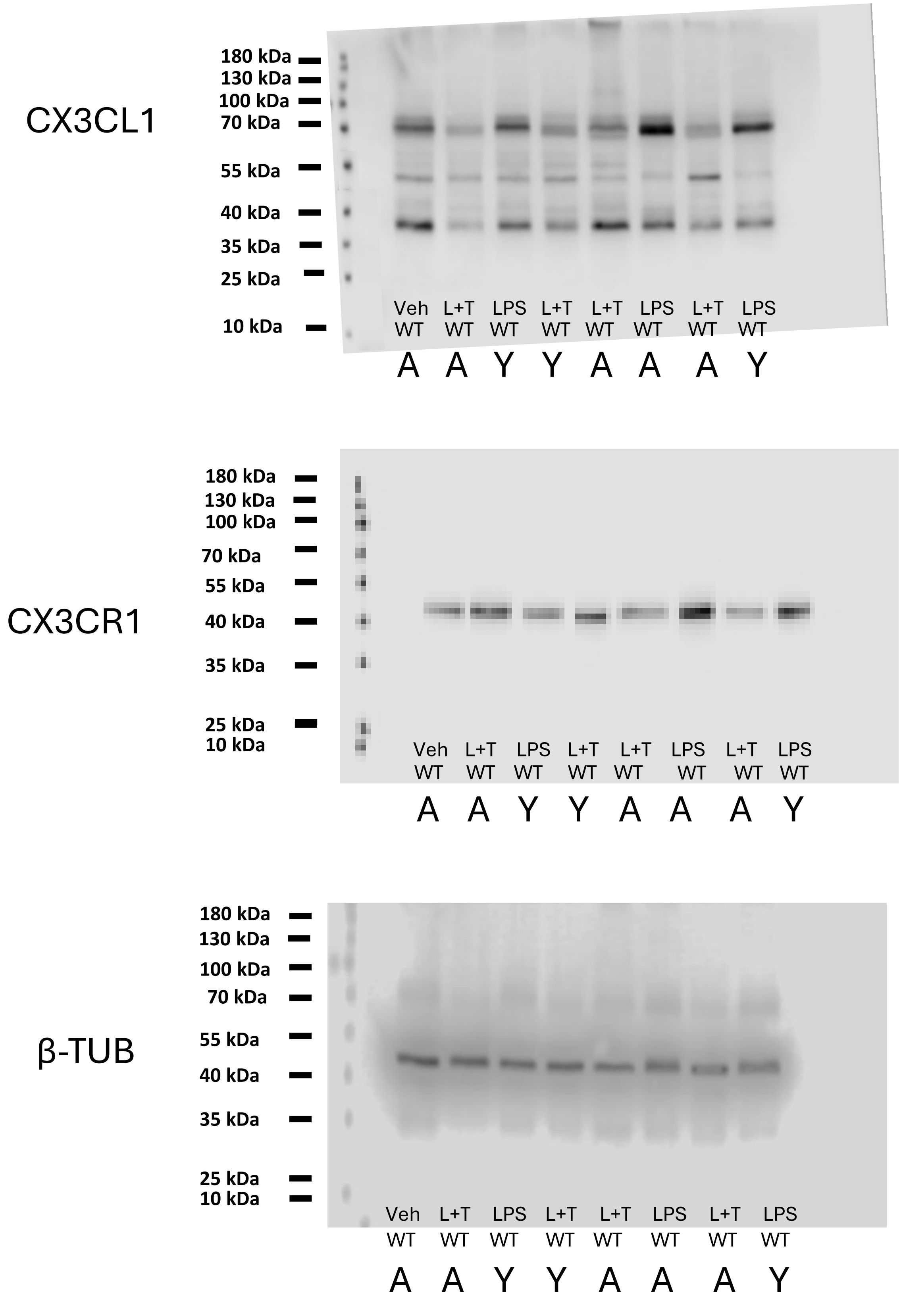
CX3CL1
180 kDa
130 kDa
100 kDa
70 kDa
55 kDa
40 kDa
35 kDa
25 kDa
10 kDa
Veh WT
L+T WT
LPS WT
L+T WT
L+T WT
LPS WT
L+T WT
LPS WT
A A Y Y A A A Y
CX3CR1
180 kDa
130 kDa
100 kDa
70 kDa
55 kDa
40 kDa
35 kDa
25 kDa
10 kDa
Veh WT
L+T WT
LPS WT
L+T WT
L+T WT
LPS WT
L+T WT
LPS WT
A A Y Y A A A Y
β-TUB
180 kDa
130 kDa
100 kDa
70 kDa
55 kDa
40 kDa
35 kDa
25 kDa
10 kDa
Veh WT
L+T WT
LPS WT
L+T WT
L+T WT
LPS WT
L+T WT
LPS WT
A A Y Y A A A Y

Fig 5

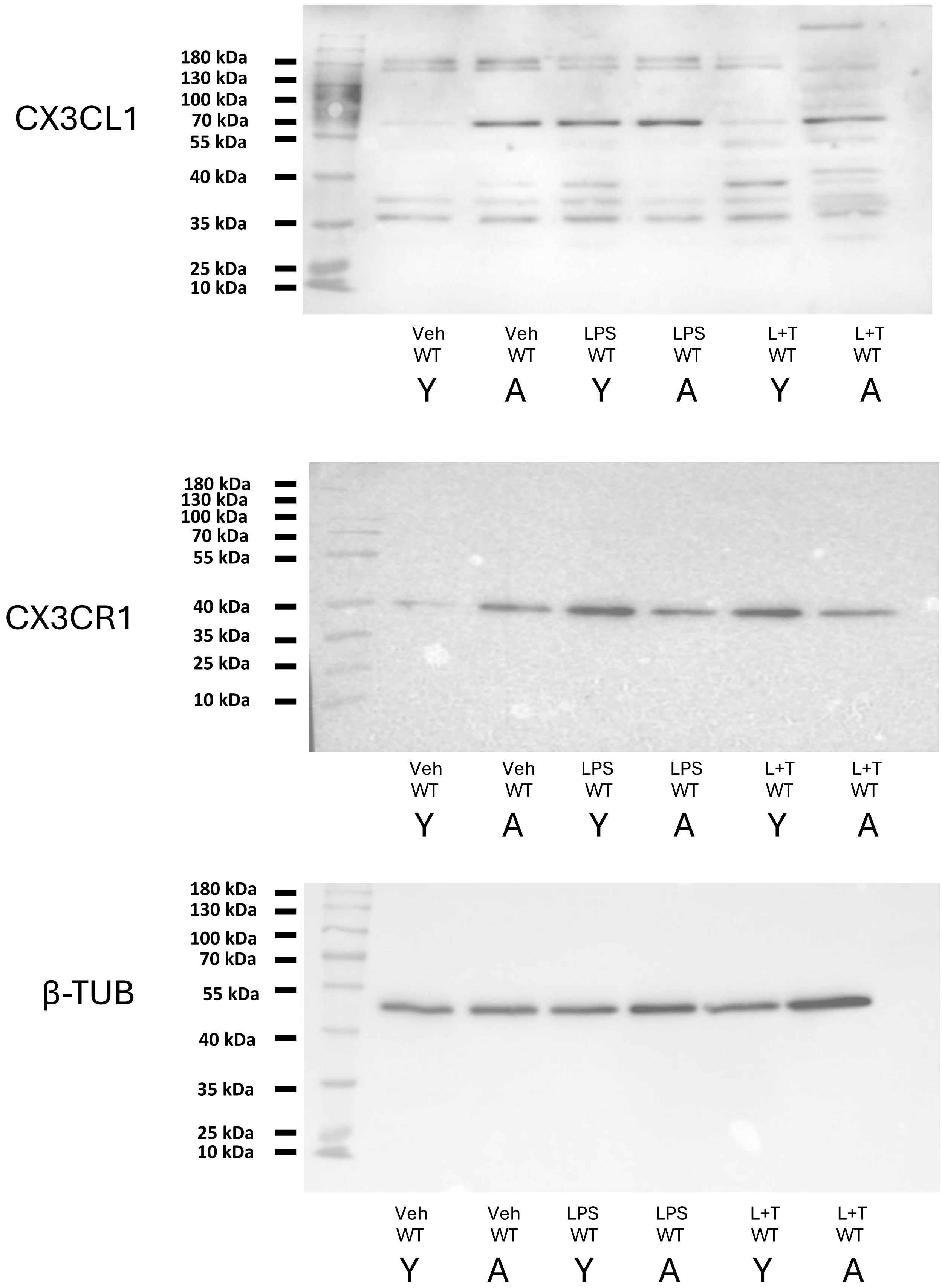

CX3CL1
180 kDa
130 kDa
100 kDa
70 kDa
55 kDa
40 kDa
35 kDa
25 kDa
10 kDa
Veh WT Y
Veh WT A
LPS WT Y
LPS WT A
L+T WT Y
L+T WT A
CX3CR1
180 kDa
130 kDa
100 kDa
70 kDa
55 kDa
40 kDa
35 kDa
25 kDa
10 kDa
Veh WT Y
Veh WT A
LPS WT Y
LPS WT A
L+T WT Y
L+T WT A
β-TUB
180 kDa
130 kDa
100 kDa
70 kDa
55 kDa
40 kDa
35 kDa
25 kDa
10 kDa
Veh WT Y
Veh WT A
LPS WT Y
LPS WT A
L+T WT Y
L+T WT A

Fig 5

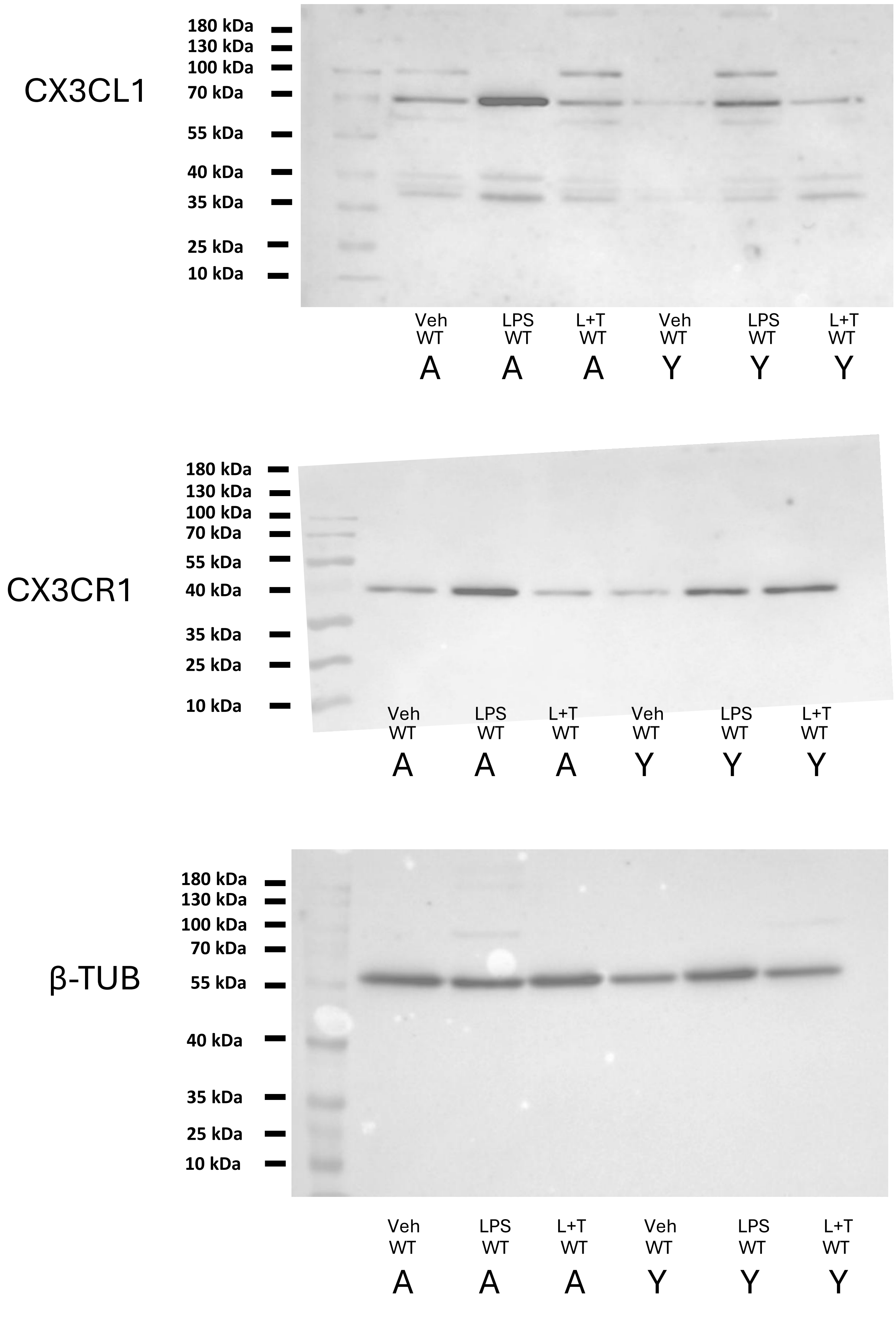
CX3CL1
180 kDa
130 kDa
100 kDa
70 kDa
55 kDa
40 kDa
35 kDa
25 kDa
10 kDa
Veh WT A
LPS WT A
L+T WT A
Veh WT Y
LPS WT Y
L+T WT Y
CX3CR1
180 kDa
130 kDa
100 kDa
70 kDa
55 kDa
40 kDa
35 kDa
25 kDa
10 kDa
Veh WT A
LPS WT A
L+T WT A
Veh WT Y
LPS WT Y
L+T WT Y
β-TUB
180 kDa
130 kDa
100 kDa
70 kDa
55 kDa
40 kDa
35 kDa
25 kDa
10 kDa
Veh WT A
LPS WT A
L+T WT A
Veh WT Y
LPS WT Y
L+T WT Y

Fig 5

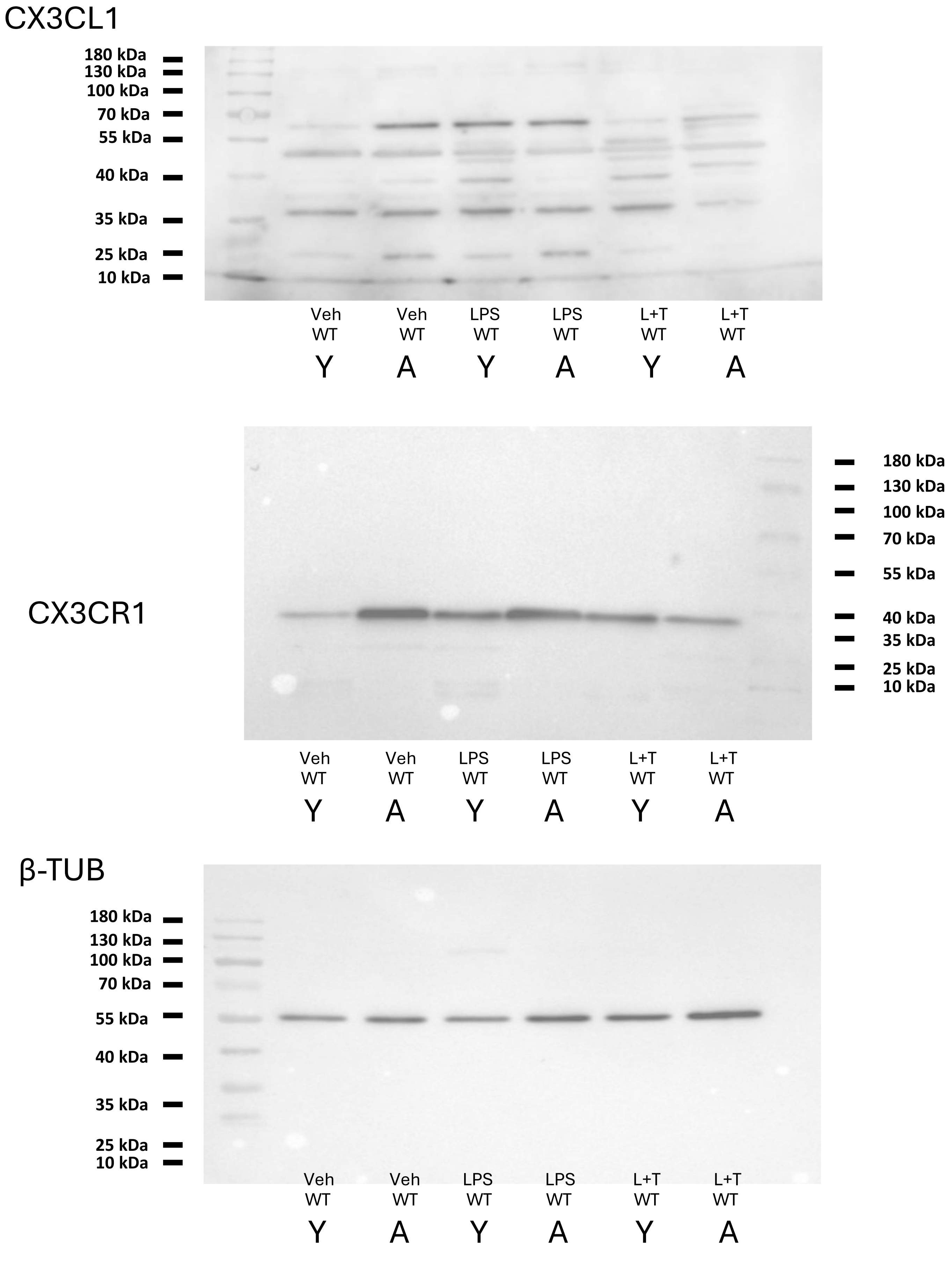
CX3CL1
180 kDa
130 kDa
100 kDa
70 kDa
55 kDa
40 kDa
35 kDa
25 kDa
10 kDa
Veh WT Y
Veh WT A
LPS WT Y
LPS WT A
L+T WT Y
L+T WT A
CX3CR1
180 kDa
130 kDa
100 kDa
70 kDa
55 kDa
40 kDa
35 kDa
25 kDa
10 kDa
Veh WT Y
Veh WT A
LPS WT Y
LPS WT A
L+T WT Y
L+T WT A
β-TUB
180 kDa
130 kDa
100 kDa
70 kDa
55 kDa
40 kDa
35 kDa
25 kDa
10 kDa
Veh WT Y
Veh WT A
LPS WT Y
LPS WT A
L+T WT Y
L+T WT A
